# Sodium-Glucose Cotransporter 2 Inhibitors, Erythrocytosis, and Thrombosis in Adults With Type 2 Diabetes

**DOI:** 10.1001/jamanetworkopen.2025.17086

**Published:** 2025-06-23

**Authors:** Maor Lewis, Nitzan Burrack, Anthony Heymann, Alon Grossman, Tsipora Neuman, Ran Abuhasira

**Affiliations:** 1 Department of Family Medicine, Meuhedet Health Maintenance Organization, Tel-Aviv, Israel; 2 Faculty of Medical and Health Sciences, Tel-Aviv University, Tel-Aviv, Israel; 3 Clinical Research Center, Soroka University Medical Center, Be’er Sheva, Israel; 4 Faculty of Health Sciences, Ben-Gurion University of the Negev, Be’er Sheva, Israel; 5 Department of Internal Medicine B, Rabin Medical Center, Beilinson Campus, Petah Tikva, Israel; 6 Division of Hematology, Sourasky Medical Center, Tel-Aviv, Israel

## Abstract

**Importance:**

Sodium-glucose cotransporter 2 inhibitors (SGLT2is) are widely used and have been shown to induce erythrocytosis. However, the clinical implications of this phenomenon, particularly its association with venous and arterial thrombotic events, remain uncertain.

**Objective:**

To assess the extent, temporal course, and thrombotic complications of SGLT2i-induced erythrocytosis.

**Design, Setting, and Participants:**

This retrospective cohort study used patient data from Israel’s largest health care organization from January 1, 2015, through June 30, 2024. Adult patients aged 18 years or older with type 2 diabetes who initiated SGLT2is were identified and compared with those who initiated dipeptidyl peptidase 4 inhibitors (DPP-4is) or glucagon-like peptide 1 receptor agonists (GLP-1RAs) using a propensity score–matched, active-comparator, new-user cohort design.

**Exposure:**

New initiation of an SGLT2i, a GLP-1RA, or a DPP-4i.

**Main Outcomes and Measures:**

Outcomes included erythrocytosis within 1 year of initiating SGLT2is, as well as arterial and venous thrombotic events until the end of follow-up. Prevalence rates, rate differences, and odds ratios (ORs) for new-onset erythrocytosis and hemoglobin increase greater than 0.5 g/dL with 95% CIs were calculated. Hazard ratios (HRs) and 95% CIs for thrombotic outcomes were estimated using time-varying Cox proportional hazards regression models.

**Results:**

After 1:1 propensity score matching, a total of 137 552 adults were included in the SGLT2i vs DPP-4i cohort (68 776 pairs; SGLT2i initiators: mean [SD] age, 64.55 [12.03] years; 55.3% male; DPP-4i initiators: mean [SD] age, 64.73 [13.08] years; 53.5% male) and 131 512 adults in the SGLT2i vs GLP-1RA cohort (65 756 pairs; SGLT2i initiators: mean [SD] age, 63.73 [11.87] years; 52.0% male; GLP-1RA initiators: mean [SD] age, 62.77 [11.56] years; 51.4% female). Among SGLT2i initiators, erythrocytosis prevalence in the SGLT2i vs DPP-4i cohort increased by 5.5% (95% CI, 5.1%-5.8%) and in the SGLT2i vs GLP-1RA cohort by 5.8% (95% CI, 5.4%-6.2%). In the SGLT2i vs GLP-1RA cohort, hemoglobin increased by 0.37 g/dL (95% CI, 0.36-0.38 g/dL) and hematocrit by 1.50% (95% CI, 1.48%-1.53%). Male sex (adjusted OR [AOR], 4.12; 95% CI, 3.80-4.48), smoking (AOR, 2.00; 95% CI, 1.85-2.16), and the use of empagliflozin vs dapagliflozin (AOR, 1.16; 95% CI, 1.09-1.25) were associated with an increased risk of developing erythrocytosis. New-onset erythrocytosis was not associated with an increased risk of myocardial infarction (HR, 0.92; 95% CI, 0.58-1.44), venous thromboembolism (HR, 1.56; 95% CI, 0.68-3.60), or stroke (HR, 1.26; 95% CI, 0.84-1.90).

**Conclusions and Relevance:**

In this cohort study of patients with type 2 diabetes, SGLT2i use was associated with a higher risk of erythrocytosis compared with DPP-4is and GLP-1RAs; however, erythrocytosis was not associated with thrombotic events. These findings provide important reassurance regarding the safety of SGLT2i-induced erythrocytosis.

## Key Points

**Question:**

What is the association of sodium-glucose cotransporter 2 inhibitors (SGLT2is) with erythrocytosis and thrombosis?

**Findings:**

In this cohort study of 269 064 propensity score–matched adult patients with type 2 diabetes, SGLT2i initiation was associated with a higher prevalence of erythrocytosis compared with dipeptidyl peptidase 4 inhibitors and glucagon-like peptide 1 receptor agonists. Erythrocytosis was not associated with an increased risk of arterial or venous thrombotic events.

**Meaning:**

These findings suggest that SGLT2i-induced erythrocytosis is prevalent but not associated with thrombotic risk.

## Teaser

This cohort study evaluates the prevalence, temporal trends, and thrombotic complications of sodium-glucose cotransporter 2 inhibitor (SGLT2i)–induced erythrocytosis among adult patients with type 2 diabetes.

## Introduction

Sodium-glucose cotransporter 2 inhibitors (SGLT2is) are widely used glucose-lowering medications for the treatment of type 2 diabetes, heart failure, and chronic kidney disease. These agents have demonstrated beneficial effects on glycemic control, cardiovascular outcomes, heart failure hospitalizations, and the progression to end-stage kidney disease.^1,2,3,4,5,6^

Over the past decade, the widespread use of SGLT2is in clinical practice has led to increased awareness of SGLT2i-induced erythrocytosis. Treatment with SGLT2is has been shown to increase hematocrit by 2% to 5% and raise hemoglobin levels by 0.4 to 1.0 g/dL compared with placebo across all SGLT2is,^3,7,8,9,10,11^ with empagliflozin showing the highest increase in hematocrit^3,7,12^ and dapagliflozin being the only SGLT2i with a dose-dependent effect.^12^ The exact mechanism behind SGLT2i-induced erythrocytosis remains unclear. Although initially attributed to hemoconcentration from diuretic effects, the more widely accepted mechanism is increased erythropoiesis.^9,13,14^

Studies in the general population have shown that elevated hemoglobin and hematocrit levels are associated with an increased risk of arterial and venous thrombotic events.^15,16^ A meta-analysis of randomized clinical trials and large observational studies did not find an association between SGLT2i use and venous thromboembolism.^17,18,19,20^ However, evidence remains insufficient to evaluate the association between SGLT2i use and thrombotic events in patients with SGLT2i-induced erythrocytosis.^9^ One of the few reports available included 100 patients with type 2 diabetes. Over a 2-year follow-up, 10 thrombotic events were recorded (7 arterial and 3 venous), which were not associated with baseline or peak hemoglobin or hematocrit levels and occurred independently of phlebotomy.^21^ In fact, higher hematocrit and hemoglobin levels have been associated with more favorable cardiovascular and kidney outcomes in several studies.^12,22,23^ The SGLT2is have also been suggested for the prevention or correction of anemia in patients with type 2 diabetes.^10,24^

The aim of our study was to evaluate the prevalence, risk factors, and temporal trends of SGLT2i-induced erythrocytosis and the extent of hemoglobin and hematocrit increases after SGLT2i initiation in a nationwide cohort of patients with type 2 diabetes in Israel. Additionally, we aimed to assess whether SGLT2i-induced erythrocytosis is associated with an increased risk of arterial and venous thrombosis.

## Methods

### Source Population

This retrospective cohort study was conducted using electronic medical records from Clalit Health Services (CHS), the largest health care organization in Israel that serves approximately 5.4 million people, representing 52% of the country’s population. The CHS owns and operates 1500 primary care clinics and 14 hospitals, accounting for 30% of Israel’s acute care hospital beds, and its administrative and clinical database integrates inpatient, outpatient, laboratory, and pharmacy data. The Rabin Medical Center Institutional Research Review Board approved the study and granted a waiver of informed consent as all patient records were anonymized and deidentified prior to analysis. Data were extracted using the Clalit Research Data Sharing Platform powered by MDClone. Our study followed the Strengthening the Reporting of Observational Studies in Epidemiology (STROBE) reporting guideline for cohort studies.

### Study Population and Design

We conducted a propensity score–matched, active-comparator, new-user cohort study to compare the risk of erythrocytosis and changes in hemoglobin levels in patients who initiated SGLT2is or a comparator, including dipeptidyl peptidase 4 inhibitor (DPP-4i) or glucagon-like peptide 1 receptor agonist (GLP-1RA). We included adult patients aged 18 years or older with type 2 diabetes who were prescribed SGLT2is, DPP-4is, or GLP-1RAs for the first time after at least 1 year of continuous enrollment in CHS. The study period spanned from January 1, 2015, through June 30, 2024. Medication adherence was assessed using pharmacy claims data, and patients were considered treated with the medication for 60 days after their last consecutive prescription purchase. We assumed that all patients were taking 1 pill of SGLT2i or DPP-4i per day and daily or weekly injections for GLP-1RAs. We excluded patients without at least 1 complete blood count in the year prior to the index date, who had ever received a diagnosis of familial polycythemia or myeloproliferative disorders (polycythemia vera, essential thrombocythemia, and myelofibrosis), and with a diagnosis of secondary erythrocytosis in the year prior to the index date.

### Outcome Assessment and Follow-Up

We used 2 different definitions to assess the occurrence of erythrocytosis in the first year following the index date. The first definition followed the 2005 British Society of Hematology criteria, which define hemoglobin concentration as greater than 18.5 g/dL or hematocrit of at least 52% in men and hemoglobin concentration greater than 16.5 g/dL or hematocrit of at least 48% in women (referred to as strict criteria).^25^ The second definition was based on the 2016 World Health Organization classification, which defines hemoglobin concentration as greater than 16.5 g/dL or hematocrit greater than 49% in men and hemoglobin concentration greater than 16.0 g/dL or hematocrit greater than 48% in women (referred to as lenient criteria).^26^ To convert hemoglobin values to grams per liter, multiply by 10; to convert hematocrit values to a proportion of 1.0, multiply by 0.01. For both definitions, we used the maximum hemoglobin value of each patient in the year prior to and following the index date. To avoid acute effects of bleeding and other acute illnesses, we only used hemoglobin values from community clinic records and not from hospitalizations or emergency department visits.

Additional outcomes included the development of thrombotic events, including arterial (myocardial infarction and stroke) and venous (deep vein thrombosis and pulmonary embolism), which were defined using *International Classification of Diseases, Ninth Revision* and *International Statistical Classification of Diseases, Tenth Revision* codes (eTable 11 in Supplement 1). Subgroup analyses included the type of SGLT2i used, sex, smoking status, and timing of erythrocytosis from the index date (0-3 months, 3-6 months, 6-9 months, and 9-12 months). The population sector (Jewish or Arab) was based on CHS’s administrative classification of the primary clinic in which each participant was registered and included to describe the demographic characteristics of the study population. In this analysis, we also used the maximum hemoglobin value for each patient in each period to assess erythrocytosis. Follow-up began on the date of first dispensation (index date) and continued until the end of the study period (June 30, 2024), CHS membership termination, occurrence of a study outcome, a switch to or addition of the comparator medication, or death, whichever occurred first.

### Statistical Analysis

We used 3 patient cohorts in this study: (1) SGLT2i vs DPP-4i, (2) SGLT2i vs GLP-1RA, and (3) only SGLT2i (unmatched cohort). For cohorts 1 and 2, we performed a propensity score–matched, active-comparator, new-user cohort study to compare the risk of erythrocytosis in patients with prevalent type 2 diabetes initiating SGLT2is vs DPP-4is and SGLT2is vs GLP-1RAs (eMethods in Supplement 1). We used Cox proportional hazard regression models after propensity score matching and further adjustment for demographics and clinical covariates selected a priori based on clinical significance (sex, age, smoking status, ischemic heart disease, hypertension, and estimated glomerular filtration rate) to obtain hazard ratios (HRs) for the first occurrence of erythrocytosis (lenient criteria), myocardial infarction, stroke, deep venous thrombosis, and pulmonary embolism (categorized as venous thromboembolism) within 1 year from the index date. For the outcome of erythrocytosis, patients with erythrocytosis prior to the index date were excluded from that analysis.

For cohort 3, the unmatched population treated with SGLT2is, we used a Cox proportional hazards regression model to assess thrombotic outcomes with follow-up of 1 year from the index date. The model included new-onset erythrocytosis (conducted both with lenient and strict criteria) as an independent time-varying variable, with adjustments for sex, age, smoking status, SGLT2i type, estimated glomerular filtration rate, and the comorbidities included in the propensity score model. The proportional hazards assumption was tested and satisfied for each variable by comparing survival curves and performing the Schoenfeld global test. In cohort 3, we performed Kaplan-Meier analysis with log-rank testing to assess the cumulative incidence of thrombotic events, including myocardial infarction, venous thromboembolism, and stroke, stratified by the development of new-onset erythrocytosis and follow-up until the end of the study period.

In cohort 3, we used multivariable logistic regression to assess factors associated with the risk of developing erythrocytosis according to the lenient criteria. We excluded patients with erythrocytosis prior to the index date from this analysis. We also used multivariable logistic regression to assess factors associated with the absolute increase in mean hemoglobin of greater than 0.5 g/dL after initiating SGLT2is. The variables in the models were chosen based on clinical significance and univariable significance (*P* < .05).

For all the cohorts, we assessed the unadjusted rate difference with the 95% CI of hemoglobin levels (grams per deciliter), hematocrit levels (percent), and erythrocytosis rates before and after the treatments. For cohorts 1 and 2, after propensity score matching, we compared hemoglobin and hematocrit levels only for patients with blood counts both before and after the index date. For all the cohorts, we used locally weighted scatterplot smoothing curves to graph the association between hemoglobin or erythrocytosis percentages over time, before and after the index date, using a span of 0.3.^27^ Estimated glomerular filtration rate was calculated using the 2009 Chronic Kidney Disease Epidemiology Collaboration equation.^28^ The analyses were performed using R, version 4.2.3 (R Foundation for Statistical Computing). The threshold for statistical significance was *P* < .05.

## Results

### Study Population and Baseline Characteristics

We identified 161 464 individuals who met the inclusion and exclusion criteria for the SGLT2i vs DPP-4i cohort, including 86 468 SGLT2i initiators (mean [SD] age, 64.5 [12.0] years; 41.3% female and 58.7% male; 27.8% of Arab and 72.2% Jewish ethnicity) and 74 996 DPP-4i initiators (mean [SD] age, 65.3 [13.0] years, 48.0% female and 52.0% male; 19.4% of Arab and 80.6% Jewish ethnicity) (eFigure 1 in Supplement 1). For the SGLT2i vs GLP-1RA cohort, 198 651 patients met the study inclusion and exclusion criteria, including 114 436 SGLT2i initiators (mean [SD] age, 66.2 [11.6] years; 39.0% female and 61.0% male; 19.8% of Arab and 80.2% Jewish ethnicity) and 84 215 GLP-1RA initiators (mean [SD] age, 59.9 [12.8] years; 57.0% female and 43.0% male; 32.9% of Arab and 67.1% Jewish ethnicity) (eFigure 2 in Supplement 1).

After 1:1 propensity score matching for cohorts 1 and 2, the DPP-4i vs SGLT2i group consisted of 68 776 pairs (SGLT2i initiators: mean [SD] age, 64.55 [12.03] years; 44.7% female and 55.3% male; 23.5% of Arab and 73.1% Jewish ethnicity; DPP-4i initiators: mean [SD] age, 64.73 [13.08] years; 46.5% female and 53.5% male; 19.9% of Arab and 77.2% Jewish ethnicity), and the GLP-1RA vs SGLT2i group consisted of 65 756 pairs (SGLT2i initiators: mean [SD] age, 63.73 [11.87] years; 48.0% female and 52.0% male; 22.7% of Arab and 74.4% Jewish ethnicity; GLP-1RA initiators: mean [SD] age, 62.77 [11.56] years; 51.4% female and 48.6% male; 28.1% of Arab and 68.6% Jewish ethnicity). The groups were well balanced across most baseline characteristics (Table 1; eTables 1 and 2 in Supplement 1).

**Table 1.** Baseline Characteristics in the 1:1 Propensity Score–Matched Population With Type 2 Diabetes Initiating SGLT2is vs DPP-4is or GLP-1RAs

Characteristic	Cohort 1, SGLT2is vs DPP-4is			Cohort 2, SGLT2is vs GLP-1RAs		
	DPP-4i (n = 68 776)	SGLT2i (n = 68 776)	SMD (95% CI)	GLP-1RA (n = 65 756)	SGLT2i (n = 65 756)	SMD (95% CI)
Age, mean (SD), y	64.73 (13.08)	64.55 (12.03)	0.01 (0.00 to 0.02)	62.77 (11.56)	63.73 (11.87)	−0.08 (−0.09 to −0.07)
Sex, No. (%)						
Female	31 986 (46.5)	30 723 (44.7)	0.04 (0.03 to 0.05)	33 831 (51.4)	31 561 (48.0)	0.07 (0.06 to 0.08)
Male	36 790 (53.5)	38 053 (55.3)	0.04 (0.03 to 0.05)	31 925 (48.6)	34 195 (52.0)	
Ethnicity, No. (%)						
Arab	13 661 (19.9)	16 162 (23.5)	0.09 (0.08 to 0.10)	18 452 (28.1)	14 909 (22.7)	0.13 (0.12 to 0.14)
Jewish	53 072 (77.2)	50 291 (73.1)		45 088 (68.6)	48 950 (74.4)	
Missing	2043 (3.0)	2323 (3.4)		2216 (3.4)	2257 (3.4)	
Socioeconomic status, No. (%)						
Very high	4477 (6.5)	4370 (6.4)	0.06 (0.05 to 0.07)	3950 (6.0)	4167 (6.3)	0.05 (0.04 to 0.06)
High	15 365 (22.3)	15 092 (21.9)		13 514 (20.6)	14 166 (21.5)	
Medium	23 552 (34.2)	22 683 (33.0)		20 666 (31.4)	21 256 (32.3)	
Low	19 470 (28.3)	19 656 (28.6)		19 744 (30.0)	18 808 (28.6)	
Very low	2122 (3.1)	2771 (4.0)		3637 (5.5)	3388 (5.2)	
Missing	3790 (5.5)	4204 (6.1)		4245 (6.5)	3971 (6.0)	
Smoking, No. (%)						
Never	42 326 (61.5)	41 335 (60.1)	0.03 (0.02 to 0.04)	39 918 (60.7)	39 314 (59.8)	0.02 (0.01 to 0.03)
Past	15 166 (22.1)	15 915 (23.1)		15 153 (23.0)	15 601 (23.7)	
Current	11 284 (16.4)	11 526 (16.8)		10 685 (16.2)	10 841 (16.5)	
Comorbidities, No. (%)						
Obesity	43 913 (63.8)	46 418 (67.5)	−0.08 (−0.09 to −0.07)	55 892 (85.0)	52 574 (80.0)	0.13 (0.12 to 0.14)
Hypertension	47 661 (69.3)	49 084 (71.4)	−0.05 (−0.06 to −0.03)	46 450 (70.6)	47 177 (71.7)	−0.02 (−0.04 to −0.01)
Myocardial infarction	6447 (9.4)	8258 (12.0)	−0.09 (−0.10 to −0.07)	5326 (8.1)	7241 (11.0)	−0.10 (−0.11 to −0.09)
Stroke	6462 (9.4)	6803 (9.9)	−0.02 (−0.03 to −0.01)	5787 (8.8)	6101 (9.3)	−0.02 (−0.03 to −0.01)
Ischemic heart disease	17 762 (25.8)	20 800 (30.2)	−0.10 (−0.11 to −0.09)	14 948 (22.7)	17 865 (27.2)	−0.10 (−0.11 to −0.09)
Heart failure	7128 (10.4)	8812 (12.8)	−0.08 (−0.09 to −0.07)	6091 (9.3)	7769 (11.8)	−0.08 (−0.09 to −0.07)
VTE	3118 (4.5)	3367 (4.9)	−0.02 (−0.03 to −0.01)	3502 (5.3)	3413 (5.2)	0.01 (0.00 to 0.02)
Atrial fibrillation	6660 (9.7)	7424 (10.8)	−0.04 (−0.05 to −0.03)	5443 (8.3)	6403 (9.7)	−0.05 (−0.06 to −0.04)
Chronic kidney disease	10 999 (16.0)	10 395 (15.1)	0.02 (0.01 to 0.03)	8935 (13.6)	9150 (13.9)	−0.01 (−0.02 to 0.00)
Peripheral vascular disease	5276 (7.7)	5487 (8.0)	−0.01 (−0.02 to 0.00)	4301 (6.5)	4760 (7.2)	−0.03 (−0.04 to −0.02)
Obstructive lung diseases	9946 (14.5)	10 385 (15.1)	−0.02 (−0.03 to −0.01)	10 488 (15.9)	10 278 (15.6)	0.01 (0.00 to 0.02)
Obstructive sleep apnea	840 (1.2)	993 (1.4)	−0.02 (−0.03 to −0.01)	1263 (1.9)	1220 (1.9)	0.00 (−0.01 to 0.02)
Liver diseases	12 735 (18.5)	13 700 (19.9)	−0.04 (−0.05 to −0.03)	15 236 (23.2)	14 697 (22.4)	0.02 (0.01 to 0.03)
Cancer	9468 (13.8)	9345 (13.6)	0.01 (−0.01 to 0.02)	8089 (12.3)	8495 (12.9)	−0.02 (−0.03 to −0.01)
Laboratory results^a^						
HbA_1c_, mean (SD), %	8.16 (1.67)	8.08 (1.72)	0.04 (0.03 to 0.06)	8.23 (1.84)	8.10 (1.67)	0.07 (0.06 to 0.08)
Creatinine, mean (SD), mg/dL	0.92 (0.55)	0.88 (0.30)	0.09 (0.08 to 0.10)	0.87 (0.41)	0.87 (0.30)	0.00 (−0.01 to 0.01)
eGFR, mean (SD), mL/min/1.73 m^2^	83.02 (24.16)	83.19 (21.49)	−0.01 (−0.02 to 0.00)	85.64 (22.32)	84.35 (21.45)	0.06 (0.05 to 0.07)
Hemoglobin, mean (SD), g/dL	13.45 (1.65)	13.48 (1.64)	−0.02 (−0.03 to −0.01)	13.48 (1.59)	13.47 (1.62)	0.01 (−0.01 to 0.02)
Days to first CBC after index date, median (IQR)	107.38 (55.32 to 183.32)	103.30 (52.39, 173.31)	0.06 (0.05 to 0.07)	104.38 (56.44, 174.38)	105.32 (54.40, 178.34)	−0.01 (−0.02 to 0.00)
No. of hospitalizations, mean (SD)^b^	0.37 (0.92)	0.40 (0.92)	−0.03 (−0.04 to −0.02)	0.31 (0.81)	0.35 (0.86)	−0.05 (−0.06 to −0.04)
Diabetes duration, mean (SD), y	10.45 (7.96)	10.71 (7.37)	−0.03 (−0.04 to −0.02)	10.74 (7.32)	11.14 (7.48)	−0.05 (−0.07 to −0.04)
Metformin use, No. (%)^c^	60 229 (87.6)	59 326 (86.3)	0.04 (0.03 to 0.05)	40 976 (62.3)	41 905 (63.7)	−0.03 (−0.04 to −0.02)

Abbreviations: CBC, complete blood count; DPP-4i, dipeptidyl peptidase 4 inhibitor; eGFR, estimated glomerular filtration rate; GLP-1RA, glucagon-like peptide 1 receptor agonist; HbA_1c_, hemoglobin A_1c_; SGLT2i, sodium-glucose cotransporter 2 inhibitor; SMD, standardized mean difference; VTE, venous thromboembolism.

^a^ Most recent value in the year preceding the index date. To convert HbA_1c_ to proportion of hemoglobin, multiply by 0.01; to convert creatinine to micromoles per liter, multiply by 76.25; to convert hemoglobin to grams per liter, multiply by 10.

^b^ Frequency during the previous year.

^c^ Defined as patients who purchased metformin at least 3 times in the year preceding the index date.

### Erythrocytosis Rates

In the SGLT2i vs DPP-4i propensity score–matched cohort, before treatment initiation, the prevalence of erythrocytosis (lenient criteria) was 6.7% (4601 of 68 775 patients) in the SGLT2i group and 5.6% (3863 of 68 721 patients) in the DPP-4i group. Following treatment, the prevalence of erythrocytosis increased markedly in the SGLT2i group, rising to 12.1% (5707 of 46 993 patients) (difference, 5.5%; 95% CI, 5.1%-5.8%). In contrast, the prevalence decreased in the DPP-4i group to 3.5% (1564 of 44 958 patients) (difference, −2.1%; 95% CI, −2.4% to −1.9%) (Table 2; eTable 5 and eFigure 5 in Supplement 1). Similar trends were observed when applying strict erythrocytosis criteria, with a 2.6% (95% CI, 2.3%-2.8%) increase in the SGLT2i group (from 1.8% to 4.3%) compared with a minimal decrease in the DPP-4i group (from 1.4% to 0.9%; difference, −0.5%; 95% CI, −0.6% to −0.3%). Erythrocytosis rates before and after initiating SGLT2is in the unmatched cohort (cohort 3) were similar to those observed in the propensity score–matched cohorts (eTables 3 and 4 in Supplement 1).

**Table 2.** Erythrocytosis Rates Among Patients With Type 2 Diabetes Before and After Initiating SGLT2is vs DPP-4is or GLP-1RAs, After Propensity Score Matching

Group characteristic^a^	Comparator			SGLT2i		
	Before, No. (%)	After, No. (%)	Difference, % (95% CI)	Before, No. (%)	After, No. (%)	Difference, % (95% CI)
**DPP-4i**						
All patients, No.	68 721	44 958	NA	68 775	46 993	NA
Erythrocytosis (lenient criteria)	3863 (5.6)	1564 (3.5)	−2.1 (−2.4 to −1.9)	4601 (6.7)	5707 (12.1)	5.5 (5.1 to 5.8)
Erythrocytosis (strict criteria)	945 (1.4)	416 (0.9)	−0.5 (−0.6 to −0.3)	1206 (1.8)	2024 (4.3)	2.6 (2.3 to 2.8)
Men						
Erythrocytosis (lenient criteria)	3416 (9.3)	1373 (5.8)	−3.5 (−3.9 to −3.0)	4093 (10.8)	4901 (18.9)	8.1 (7.6 to 8.7)
Erythrocytosis (strict criteria)	542 (1.5)	240 (1.0)	−0.5 (−0.6 to −0.3)	730 (1.9)	1252 (4.8)	2.9 (2.6 to 3.2)
Women						
Erythrocytosis (lenient criteria)	447 (1.4)	191 (0.9)	−0.5 (−0.7 to −0.3)	508 (1.7)	806 (3.8)	2.2 (1.9 to 2.5)
Erythrocytosis (strict criteria)	403 (1.3)	176 (0.8)	−0.4 (−0.6 to −0.3)	476 (1.5)	772 (3.7)	2.1 (1.8 to 2.4)
**GLP-1RA**						
All patients, No.	65 694	44 153	NA	65 756	42 664	NA
Erythrocytosis (lenient criteria)	4124 (6.3)	2264 (5.1)	−1.1 (−1.4 to −0.9)	4032 (6.1)	5092 (11.9)	5.8 (5.4 to 6.2)
Erythrocytosis (strict criteria)	1112 (1.7)	661 (1.5)	−0.2 (−0.4 to −0.0)	1076 (1.6)	1834 (4.3)	2.7 (2.4 to 2.9)
Men						
Erythrocytosis (lenient criteria)	3571 (11.2)	1934 (9.3)	−1.9 (−2.4 to −1.3)	3586 (10.5)	4312 (19.5)	9.0 (8.4 to 9.6)
Erythrocytosis (strict criteria)	603 (1.9)	355 (1.7)	−0.2 (−0.4 to 0.1)	654 (1.9)	1084 (4.9)	3.0 (2.7 to 3.3)
Women						
Erythrocytosis (lenient criteria)	553 (1.6)	330 (1.4)	−0.2 (−0.4 to −0.0)	446 (1.4)	780 (3.8)	2.4 (2.1 to 2.7)
Erythrocytosis (strict criteria)	509 (1.5)	306 (1.3)	−0.2 (−0.4 to 0.0)	422 (1.3)	750 (3.6)	2.3 (2.0 to 2.6)

Abbreviations: DPP-4i, dipeptidyl peptidase 4 inhibitor; GLP-1RA, glucagon-like peptide 1 receptor agonist; NA, not applicable; SGLT2i, sodium-glucose cotransporter 2 inhibitor.

^a^ Lenient criteria included hemoglobin greater than 16.5 g/dL or hematocrit greater than 49% in men and hemoglobin greater than 16.0 g/dL or hematocrit greater than 48% in women based on the 2016 World Health Organization classification. Strict criteria included hemoglobin greater than 18.5 g/dL or hematocrit of at least 52% in men and hemoglobin greater than 16.5 g/dL or hematocrit of at least 48% in women based on the 2005 British Society of Hematology criteria. To convert hemoglobin values to grams per liter, multiply by 10; to convert hematocrit values to a proportion of 1.0, multiply by 0.01.

In the SGLT2i vs GLP-1RA propensity score–matched cohort, before treatment initiation, the incidence of erythrocytosis (lenient criteria) was 6.1% (4032 of 65 756 patients) in the SGLT2i group and 6.3% (4124 of 65 694 patients) in the GLP-1RA group. Following treatment, the incidence of erythrocytosis increased markedly in the SGLT2i group, rising to 11.9% (5092 of 42 664 patients; difference, 5.8%; 95% CI, 5.4%-6.2%). In contrast, the incidence slightly decreased in the GLP-1RA group to 5.1% (2264 or 44 153 patients; difference, −1.1%; 95% CI, −1.4% to −0.9%) (Table 2; eTable 6 and eFigure 6 in Supplement 1). Similar trends were observed when applying strict erythrocytosis criteria, with a 2.7% increase in the SGLT2i group (from 1.6% to 4.3%; difference, 2.7%; 95% CI, 2.4%-2.9%) compared with a minimal decrease in the GLP-1RA group (from 1.7% to 1.5%; difference, −0.2%; 95% CI, −0.4% to 0.0%).

### Hemoglobin and Hematocrit Levels Over Time

**Figure 1.**
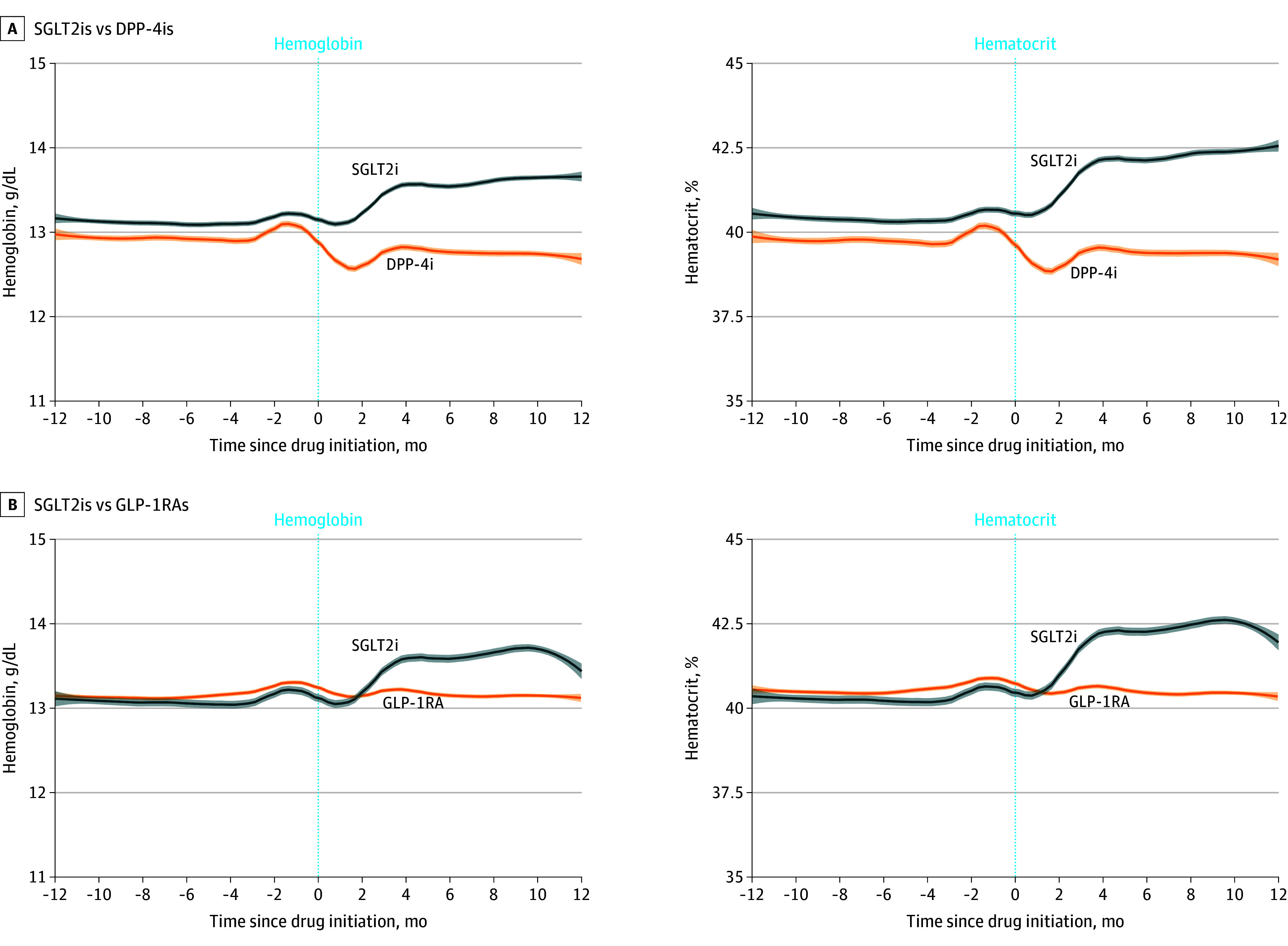
Hemoglobin and Hematocrit Levels Over Time Among Patients With Type 2 Diabetes Initiating Sodium-Glucose Cotransporter 2 Inhibitors (SGLT2is) vs Dipeptidyl Peptidase 4 Inhibitors (DPP-4is) or Glucagon-Like Peptide 1 Receptor Agonists (GLP-1RAs) After Propensity Score Matching Lines were smoothed using the locally weighted scatterplot smoothing method. The shaded areas around the curves indicate 95% CIs.

In the SGLT2i vs DPP-4i propensity score–matched cohort, the mean (SD) hemoglobin levels in the SGLT2i group increased from 13.37 (1.59) g/dL at baseline to 13.72 (1.72) g/dL 1 year after treatment (difference, 0.35 g/dL; 95% CI, 0.34-0.36 g/dL) compared with the DPP-4i group in which the mean (SD) hemoglobin levels decreased from 13.28 (1.61) g/dL at baseline to 13.09 (1.62) g/dL (difference, −0.19; 95% CI, −0.20 to −0.18 g/dL). In the SGLT2i group, mean (SD) hematocrit levels increased from 41.07% (4.42%) to 42.49% (4.85%) in the same period (difference, 1.42%; 95% CI, 1.39%-1.44%) (Figure 1A; eTable 7 and eFigure 3 in Supplement 1). In the SGLT2i vs GLP-1RA propensity score–matched cohort, the mean (SD) hemoglobin levels in the SGLT2i group increased from 13.36 (1.58) g/dL at baseline to 13.72 (1.70) g/dL 1 year after treatment (difference, 0.37 g/dL; 95% CI, 0.36-0.38 g/dL) compared with the GLP-1RA group in which the mean (SD) hemoglobin decreased minimally from 13.38 (1.56) g/dL at baseline to 13.37 (1.55) g/dL (difference, −0.01 g/dL; 95% CI, −0.02 to 0.00 g/dL). In the SGLT2i group, mean (SD) hematocrit levels increased from 41.02% (4.37%) to 42.52% (4.81%) in the same period (difference, 1.50%; 95% CI, 1.48%-1.53%) (Figure 1B; eTable 8 and eFigure 4 in Supplement 1).

### Incident Erythrocytosis and Hemoglobin Increment

In multivariable analyses of cohort 3, variables associated with incident erythrocytosis after treatment initiation were male sex (adjusted odds ratio [AOR], 4.12; 95% CI, 3.80-4.48), current smoking (AOR, 2.00; 95% CI, 1.85-2.16), and the use of empagliflozin vs dapagliflozin (AOR, 1.16; 95% CI, 1.09-1.25) (eFigures 7-9 in Supplement 1). When assessing variables associated with a mean increase in hemoglobin of greater than 0.5 g/dL after treatment initiation, male sex (AOR, 1.21; 95% CI, 1.17-1.26) and empagliflozin (AOR, 1.03; 95% CI 1.00-1.07) still showed risk but with substantially smaller ratios (Table 3). Multiple Cox proportional hazards regression showed that SGLT2is had an HR for development of erythrocytosis (lenient criteria) of 5.76 (95% CI, 5.31-6.25) compared with DPP-4is and of 3.50 (95% CI, 3.27-3.76) compared with GLP-1RAs.

**Table 3.** Association of Demographics and Comorbidities With the Development of New Erythrocytosis or With a Mean Increase in Hemoglobin Greater Than 0.5 g/dL in Patients With Type 2 Diabetes

Characteristic	New erythrocytosis		Mean increase in hemoglobin >0.5 g/dL^a^	
	Unadjusted OR (95% CI)	Adjusted OR (95% CI)	Unadjusted OR (95% CI)	Adjusted OR (95% CI)
Age	0.97 (0.97-0.98)	0.99 (0.98-0.99)	1.00 (1.00-1.00)	1.00 (1.00-1.00)
Male (vs female) sex	4.80 (4.44-5.19)	4.12 (3.80-4.48)	1.20 (1.16-1.24)	1.21 (1.17-1.26)
Smoking				
Never	1 [Reference]	1 [Reference]	1 [Reference]	1 [Reference]
Past	1.88 (1.75-2.01)	1.38 (1.28-1.48)	1.10 (1.06-1.14)	1.05 (1.01-1.09)
Current	2.96 (2.75-3.18)	2.00 (1.85-2.16)	0.97 (0.92-1.01)	0.91 (0.87-0.96)
SGLT2i group				
Dapagliflozin	1 [Reference]	1 [Reference]	1 [Reference]	1 [Reference]
Empagliflozin	1.24 (1.16-1.32)	1.16 (1.09-1.25)	1.04 (1.01-1.08)	1.03 (1.00-1.07)
Diabetes duration	0.95 (0.94-0.95)	0.97 (0.96-0.97)	1.00 (1.00-1.00)	1.00 (1.00-1.01)
Metformin use	0.94 (0.87-1.02)	0.81 (0.75-0.88)	1.05 (1.01-1.10)	1.01 (0.97-1.06)
Comorbidities				
Hypertension	0.68 (0.64-0.72)	0.98 (0.91-1.06)	0.95 (0.92-0.99)	0.98 (0.94-1.03)
Myocardial infarction	1.31 (1.22-1.40)	1.06 (0.97-1.16)	0.96 (0.92-1.00)	0.93 (0.88-0.97)
Stroke	0.74 (0.67-0.82)	0.86 (0.77-0.95)	0.91 (0.86-0.95)	0.92 (0.87-0.96)
Heart failure	0.77 (0.71-0.83)	0.87 (0.80-0.95)	0.98 (0.94-1.02)	1.05 (1.00-1.10)
VTE	0.72 (0.63-0.83)	0.94 (0.81-1.08)	0.90 (0.84-0.96)	0.92 (0.86-0.99)
Peripheral vascular disease	0.80 (0.72-0.89)	0.84 (0.75-0.93)	1.01 (0.96-1.07)	1.03 (0.97-1.09)
Obstructive lung diseases	0.80 (0.74-0.87)	0.92 (0.85-1.01)	0.95 (0.91-0.99)	0.97 (0.93-1.02)
Obstructive sleep apnea	1.13 (0.94-1.34)	1.12 (0.92-1.34)	1.13 (1.02-1.25)	1.13 (1.02-1.26)
Ischemic heart disease	1.09 (1.03-1.16)	0.91 (0.83-0.98)	0.98 (0.95-1.02)	0.98 (0.94-1.03)
Cancer	0.72 (0.66-0.79)	0.90 (0.82-0.99)	0.99 (0.94-1.03)	1.01 (0.96-1.06)
GFR stages				
1	1 [Reference]	1 [Reference]	1 [Reference]	1 [Reference]
2	0.83 (0.78-0.88)	1.07 (0.99-1.15)	0.98 (0.95-1.02)	1.00 (0.95-1.04)
3	0.47 (0.43-0.52)	0.73 (0.65-0.82)	0.91 (0.87-0.95)	0.93 (0.88-0.99)
4-5	0.22 (0.14-0.35)	0.42 (0.25-0.65)	0.68 (0.58-0.80)	0.72 (0.61-0.85)

Abbreviations: GFR, glomerular filtration rate; OR, odds ratio; SGLT2i, sodium-glucose cotransporter 2 inhibitor; VTE, venous thromboembolism.

^a^ To convert hemoglobin to grams per liter, multiply by 10.

### Association of Erythrocytosis With Thrombotic Risk

**Figure 2.**
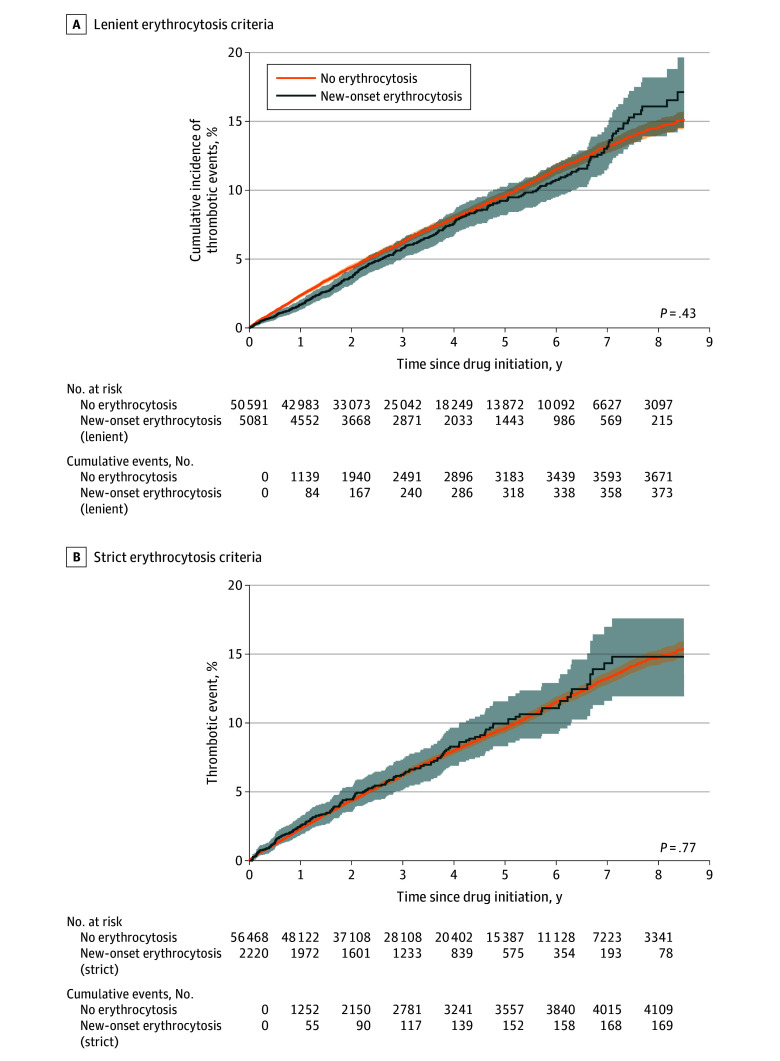
Kaplan-Meier Curves for Thrombotic Events (Myocardial Infarction, Venous Thromboembolism, and Stroke) in Sodium-Glucose Cotransporter 2 Inhibitor Initiators With and Without New-Onset Erythrocytosis Lenient criteria included hemoglobin greater than 16.5 g/dL or hematocrit greater than 49% in men and hemoglobin greater than 16.0 g/dL or hematocrit greater than 48% in women based on the 2016 World Health Organization classification. Strict criteria included hemoglobin greater than 18.5 g/dL or hematocrit of at least 52% in men and hemoglobin greater than 16.5 g/dL or hematocrit of at least 48% in women based on 2005 British Society of Hematology criteria. To convert hemoglobin values to grams per liter, multiply by 10; to convert hematocrit values to a proportion of 1.0, multiply by 0.01. The shaded areas around the curves indicate 95% CIs.

Using the strict erythrocytosis criteria as an independent variable, new-onset erythrocytosis was not associated with an increased risk of myocardial infarction (HR, 0.92; 95% CI, 0.58-1.44), venous thromboembolism (HR, 1.56; 95% CI, 0.68-3.59), or stroke (HR, 1.26; 95% CI, 0.84-1.89). Using the lenient criteria, erythrocytosis was associated with a reduced risk of stroke (HR, 0.68; 95% CI, 0.47-0.99) (eTable 10 and eFigures 10 and 11 in Supplement 1). When all thrombotic events, both arterial and venous, were considered together in Kaplan-Meier analysis, there remained no difference in risk between patients treated with SGLT2is who developed erythrocytosis and those who did not (Figure 2).

## Discussion

In this large, nationwide cohort study of adult patients with type 2 diabetes in Israel, the use of SGLT2is, compared with DPP-4is or GLP-1RAs, was associated with a higher prevalence of erythrocytosis over time, as reflected by elevated hemoglobin and hematocrit levels. These findings were consistent across a range of predefined subgroup analyses and were not influenced by the specific definition of erythrocytosis applied. Male sex, smoking, and the use of empagliflozin (vs dapagliflozin) were associated with an increased risk of developing new-onset erythrocytosis among incident users of SGLT2is. The SGLT2i-induced erythrocytosis was not associated with an increased risk of arterial and venous thrombotic events.

In our study, treatment with SGLT2is was associated with a mean increase of 0.35 and 0.37 g/dL in hemoglobin and a 1.4% and 1.5% increase in hematocrit compared with DPP-4is and GLP-1RAs, respectively. The rise in hemoglobin was slightly lower than in previous randomized clinical trials and observational studies that reported an increase in hemoglobin, ranging between 0.5 and 1.0 g/dL.^9,29^ However, a recent study from Denmark^30^ reported a mean increase of 0.43 g/dL (95% CI, 0.43-0.44 g/dL) among patients with type 2 diabetes using SGLT2is compared with DPP-4is, which is consistent with our findings. Increases in hematocrit observed in our study were lower than reported in prior meta-analyses and post hoc analyses.^7,10,12^ Additionally, a recent population-based study from Israel,^31^ which evaluated only patients with type 2 diabetes taking empagliflozin and dapagliflozin, similar to our study, reported a median hematocrit increase of 2.1%. Our lower rate (1.4%-1.5%) is unexpected, but may be explained by the lower number of men in our study.

The increase of hematocrit and hemoglobin plateaued after 3 to 4 months of treatment with SGLT2is (Figure 1; eTables 5 and 6 in Supplement 1). This timeline aligns with the increasingly accepted hypothesis that the primary mechanism underlying SGLT2i-induced erythrocytosis is increased erythropoiesis rather than hemoconcentration.^9,13,14^ Initially, the increase in hematocrit was attributed to hemoconcentration resulting from the modest diuretic effects of these drugs. However, the transient rise in urine volume, which peaks at 24 hours and returns to baseline within 1 week,^13^ did not align with the continued rise in hematocrit over 2 to 3 months.^13,14,22^ Supporting the erythropoiesis hypothesis, erythropoietin levels have been shown to rise within the first 4 weeks of SGLT2i therapy, accompanied by an increase in reticulocyte count, followed by subsequent elevations in hemoglobin and hematocrit.^14^

Male sex and smoking were associated with an increased risk of new-onset erythrocytosis. These findings may be attributed to higher baseline hematocrit levels (eFigures 7 and 8 in Supplement 1). Other covariates, particularly chronic comorbidities such as heart failure, ischemic heart disease, stroke, peripheral vascular disease, and chronic kidney disease stages 3 to 5, exhibited a reduced risk of new-onset erythrocytosis, possibly reflecting lower baseline hematocrit levels. Compared with dapagliflozin, empagliflozin showed an increased risk of developing new-onset erythrocytosis and a mean hemoglobin increase greater than 0.5 g/dL. These findings align with previous studies, including meta-analyses,^7,12,31^ and suggest that in specific circumstances, such as in a male patient with elevated baseline hematocrit levels who smokes, clinicians might prefer to initiate therapy with dapagliflozin while closely monitoring hematocrit levels. Conversely, for patients with baseline anemia, which might be corrected by treatment with SGLT2is,^10,31^ the more potent empagliflozin may prove to be more beneficial.

In our study, SGLT2i-induced erythrocytosis was not associated with an increased risk of arterial or venous thrombosis. These findings strengthen the limited existing literature^21^ that previously explored the association between SGLT2i-induced erythrocytosis and thrombotic events. Interestingly, at lower erythrocytosis thresholds, as defined in our study using the lenient criteria, there was even a reduction in the risk of stroke. This observation may be explained by previous research suggesting that increases in hematocrit and hemoglobin could serve as surrogate markers for the cardiovascular benefits associated with SGLT2i use.^12,22,23^

### Limitations

There are several limitations to our study. First, we could not entirely rule out residual confounding, though we attempted to mitigate this by adjusting for numerous clinically relevant confounders. Second, our study included only patients with type 2 diabetes, excluding those with sole indications of chronic kidney disease or heart failure, making it unclear whether the findings are generalizable to these populations. Third, due to the observational nature of the study, we were unable to assess serial hemoglobin and hematocrit measurements simultaneously for all patients. Finally, we did not account for SGLT2i dosage, which may have influenced the results, particularly for dapagliflozin for which a dose-response relationship has been previously reported.^12^

## Conclusions

In this large, nationwide, population-based cohort study of adult patients with type 2 diabetes, initiation of SGLT2is compared with GLP-1RAs and DPP-4is was associated with a higher risk of new-onset erythrocytosis and a significant increase in both hematocrit and hemoglobin levels. However, erythrocytosis was not linked to a higher risk of thrombotic events, including myocardial infarction, venous thromboembolism, and stroke. These findings provide important reassurance regarding the safety of SGLT2i-induced erythrocytosis and contribute to the limited existing literature on the subject.

## Notes

Supplement 1.
eMethods.
**eFigure 1.** Flowchart of Patients Included in the SGLT2i vs DPP-4i Cohort **eFigure 2.** Flowchart of Patients Included in the SGLT2i vs GLP-1RA Cohort **eFigure 3.** Hemoglobin (g/dL) and Hematocrit (%) Levels Over Time Among Patients With Type 2 Diabetes Initiating SGLT2is vs DPP-4is After Propensity Score Matching, Divided by Sex **eFigure 4.** Hemoglobin (g/dL) and Hematocrit (%) Levels Over Time Among Patients With Type 2 Diabetes Initiating SGLT2is vs GLP-1RAs After Propensity Score Matching, Divided by Sex **eFigure 5.** Percentage of Erythrocytosis (Lenient Criteria) Over Time Among Patients With Type 2 Diabetes Initiating SGLT2is vs DPP-4is After Propensity Score Matching, Stratified by Sex **eFigure 6.** Percentage of Erythrocytosis (Lenient Criteria) Over Time Among Patients With Type 2 Diabetes Initiating SGLT2is vs GLP-1RAs After Propensity Score Matching, Stratified by Sex **eFigure 7.** Hemoglobin (g/dL) and Hematocrit (%) Levels Over Time Among Patients With Type 2 Diabetes Initiating SGLT2is (Unmatched Cohort, Cohort 3), Divided by Sex **eFigure 8.** Hemoglobin (g/dL) and Hematocrit (%) Levels Over Time Among Patients With Type 2 Diabetes Initiating SGLT2is (Unmatched Cohort, Cohort 3), Divided by Smoking Status **eFigure 9.** Hemoglobin (g/dL) and Hematocrit (%) Levels Over Time Among Patients With Type 2 Diabetes Initiating SGLT2is (Unmatched Cohort, Cohort 3), Divided by SGLT2i Type **eFigure 10.** Kaplan-Meier Curves for MI (A), VTE (B), and Stroke (C) in SGLT2i Initiators With and Without New-Onset Erythrocytosis (Lenient Criteria) **eFigure 11.** Kaplan-Meier Curves for MI (A), VTE (B), and Stroke (C) in SGLT2i Initiators With and Without New-Onset Erythrocytosis (Strict Criteria) **eTable 1.** Baseline Characteristics of Patients With Type 2 Diabetes Initiating SGLT2is vs DPP-4is, Before and After 1:1 Propensity Score Matching **eTable 2.** Baseline Characteristics of Patients With Type 2 Diabetes Initiating SGLT2is vs GLP-1RAs, Before and After 1:1 Propensity Score Matching **eTable 3.** Erythrocytosis Rates Among Patients With Type 2 Diabetes Before and After Initiating SGLT2is (Cohort 3) **eTable 4.** Erythrocytosis Rates Among Patients With Type 2 Diabetes Before and After Initiating SGLT2is, Stratified by 3-Month Quartiles in the Year After Treatment Initiation **eTable 5.** Erythrocytosis Rates Among Patients With Type 2 Diabetes, Before and After Initiating SGLT2is vs DPP-4is, After Propensity Score Matching, Stratified by 3-Month Quartiles in the Year After Treatment Initiation **eTable 6.** Erythrocytosis Rates Among Patients With Type 2 Diabetes, Before and After Initiating SGLT2is vs GLP-1RAs, After Propensity Score Matching, Stratified by 3-Month Quartiles in the Year After Treatment Initiation **eTable 7.** Hemoglobin (g/dL) and Hematocrit (%) Levels Among Patients With Type 2 Diabetes, Before and After Initiating SGLT2is vs DPP-4is, After Propensity Score Matching **eTable 8.** Hemoglobin (g/dL) and Hematocrit (%) Levels Among Patients With Type 2 Diabetes, Before and After Initiating SGLT2is vs GLP-1RAs, After Propensity Score Matching **eTable 9.** Hemoglobin (g/dL) and Hematocrit (%) Levels Among Patients With Type 2 Diabetes Before and After Initiating SGLT2is (Unmatched Cohort), Stratified by SGLT2i Type **eTable 10. **Cox Proportional Hazards Regression Model With Time-Varying Exposure for Myocardial Infarction, Venous Thromboembolism, and Stroke Comparing Patients With Type 2 Diabetes After Initiating SGLT2is and Developing New-Onset Erythrocytosis (Cohort 3) **eTable 11.** Coding and Definitions

Supplement 2.
Data Sharing Statement
