# Supplementary material for: Sodium-Glucose Cotransporter 2 Inhibitors, Erythrocytosis, and Thrombosis in Adults With Type 2 Diabetes
Source: JAMA Netw Open. 2025 Jun 23;8(6):e2517086. doi: 10.1001/jamanetworkopen.2025.17086 (PMC12186121; doi:10.1001/jamanetworkopen.2025.17086)
Supplement: Supplement 1. — eMethods. eFigure 1. Flowchart of Patients Included in the SGLT2i vs DPP-4i Cohort eFigure 2. Flowchart of Patients Included in the SGLT2i vs GLP-1RA Cohort eFigure 3. Hemoglobin (g/dL) and Hematocrit (%) Levels Over Time Among Patients With Type 2 Diabetes Initiating SGLT2is vs DPP-4is After Propensity Score Matching, Divided by Sex eFigure 4. Hemoglobin (g/dL) and Hematocrit (%) Levels Over Time Among Patients With Type 2 Diabetes Initiating SGLT2is vs GLP-1RAs After Propensity Score Matching, Divided by Sex eFigure 5. Percentage of Erythrocytosis (Lenient Criteria) Over Time Among Patients With Type 2 Diabetes Initiating SGLT2is vs DPP-4is After Propensity Score Matching, Stratified by Sex eFigure 6. Percentage of Erythrocytosis (Lenient Criteria) Over Time Among Patients With Type 2 Diabetes Initiating SGLT2is vs GLP-1RAs After Propensity Score Matching, Stratified by Sex eFigure 7. Hemoglobin (g/dL) and Hematocrit (%) Levels Over Time Among Patients With Type 2 Diabetes Initiating SGLT2is (Unmatched Cohort, Cohort 3), Divided by Sex eFigure 8. Hemoglobin (g/dL) and Hematocrit (%) Levels Over Time Among Patients With Type 2 Diabetes Initiating SGLT2is (Unmatched Cohort, Cohort 3), Divided by Smoking Status eFigure 9. Hemoglobin (g/dL) and Hematocrit (%) Levels Over Time Among Patients With Type 2 Diabetes Initiating SGLT2is (Unmatched Cohort, Cohort 3), Divided by SGLT2i Type eFigure 10. Kaplan-Meier Curves for MI (A), VTE (B), and Stroke (C) in SGLT2i Initiators With and Without New-Onset Erythrocytosis (Lenient Criteria) eFigure 11. Kaplan-Meier Curves for MI (A), VTE (B), and Stroke (C) in SGLT2i Initiators With and Without New-Onset Erythrocytosis (Strict Criteria) eTable 1. Baseline Characteristics of Patients With Type 2 Diabetes Initiating SGLT2is vs DPP-4is, Before and After 1:1 Propensity Score Matching eTable 2. Baseline Characteristics of Patients With Type 2 Diabetes Initiating SGLT2is vs GLP-1RAs, Before and After 1:1 Propensity Score Matching eTabl [file jamanetwopen-e2517086-s001.pdf]

Supplemental Online Content

Lewis M, Burrack N, Heymann A, Grossman A, Neuman T, Abuhasira R. Sodium-glucose cotransporter 2 inhibitors, erythrocytosis, and thrombosis in adults with type 2 diabetes. *JAMA Netw Open*. 2025;8(6):e2517086. doi:10.1001/jamanetworkopen.2025.17086

eMethods.

eFigure 1. Flowchart of Patients Included in the SGLT2i vs DPP-4i Cohort

eFigure 2. Flowchart of Patients Included in the SGLT2i vs GLP-1RA Cohort

eFigure 3. Hemoglobin (g/dL) and Hematocrit (%) Levels Over Time Among Patients With Type 2 Diabetes Initiating SGLT2is vs DPP-4is After Propensity Score Matching, Divided by Sex

eFigure 4. Hemoglobin (g/dL) and Hematocrit (%) Levels Over Time Among Patients With Type 2 Diabetes Initiating SGLT2is vs GLP-1RAs After Propensity Score Matching, Divided by Sex

eFigure 5. Percentage of Erythrocytosis (Lenient Criteria) Over Time Among Patients With Type 2 Diabetes Initiating SGLT2is vs DPP-4is After Propensity Score Matching, Stratified by Sex

eFigure 6. Percentage of Erythrocytosis (Lenient Criteria) Over Time Among Patients With Type 2 Diabetes Initiating SGLT2is vs GLP-1RAs After Propensity Score Matching, Stratified by Sex

eFigure 7. Hemoglobin (g/dL) and Hematocrit (%) Levels Over Time Among Patients With Type 2 Diabetes Initiating SGLT2is (Unmatched Cohort, Cohort 3), Divided by Sex

eFigure 8. Hemoglobin (g/dL) and Hematocrit (%) Levels Over Time Among Patients With Type 2 Diabetes Initiating SGLT2is (Unmatched Cohort, Cohort 3), Divided by Smoking Status

eFigure 9. Hemoglobin (g/dL) and Hematocrit (%) Levels Over Time Among Patients With Type 2 Diabetes Initiating SGLT2is (Unmatched Cohort, Cohort 3), Divided by SGLT2i Type

eFigure 10. Kaplan-Meier Curves for MI (A), VTE (B), and Stroke (C) in SGLT2i Initiators With and Without New-Onset Erythrocytosis (Lenient Criteria)

eFigure 11. Kaplan-Meier Curves for MI (A), VTE (B), and Stroke (C) in SGLT2i Initiators With and Without New-Onset Erythrocytosis (Strict Criteria)

eTable 1. Baseline Characteristics of Patients With Type 2 Diabetes Initiating SGLT2is vs DPP-4is, Before and After 1:1 Propensity Score Matching

eTable 2. Baseline Characteristics of Patients With Type 2 Diabetes Initiating SGLT2is vs GLP-1RAs, Before and After 1:1 Propensity Score Matching

eTable 3. Erythrocytosis Rates Among Patients With Type 2 Diabetes Before and After Initiating SGLT2is (Cohort 3)

© 2025 Lewis M et al. *JAMA Network Open*.

eTable 4. Erythrocytosis Rates Among Patients With Type 2 Diabetes Before and After Initiating SGLT2is, Stratified by 3-Month Quartiles in the Year After Treatment Initiation

eTable 5. Erythrocytosis Rates Among Patients With Type 2 Diabetes, Before and After Initiating SGLT2is vs DPP-4is, After Propensity Score Matching, Stratified by 3-Month Quartiles in the Year After Treatment Initiation

eTable 6. Erythrocytosis Rates Among Patients With Type 2 Diabetes, Before and After Initiating SGLT2is vs GLP-1RAs, After Propensity Score Matching, Stratified by 3-Month Quartiles in the Year After Treatment Initiation

eTable 7. Hemoglobin (g/dL) and Hematocrit (%) Levels Among Patients With Type 2 Diabetes, Before and After Initiating SGLT2is vs DPP-4is, After Propensity Score Matching

eTable 8. Hemoglobin (g/dL) and Hematocrit (%) Levels Among Patients With Type 2 Diabetes, Before and After Initiating SGLT2is vs GLP-1RAs, After Propensity Score Matching

eTable 9. Hemoglobin (g/dL) and Hematocrit (%) Levels Among Patients With Type 2 Diabetes Before and After Initiating SGLT2is (Unmatched Cohort), Stratified by SGLT2i Type

eTable 10. Cox Proportional Hazards Regression Model With Time-Varying Exposure for Myocardial Infarction, Venous Thromboembolism, and Stroke Comparing Patients With Type 2 Diabetes After Initiating SGLT2is and Developing New-Onset Erythrocytosis (Cohort 3)

eTable 11. Coding and Definitions

This supplemental material has been provided by the authors to give readers additional information about their work.

© 2025 Lewis M et al. *JAMA Network Open*.

eMethods.

For cohorts 1 and 2, we performed a propensity score–matched, active comparator, new-user cohort study to compare the risk of erythrocytosis in patients with prevalent T2DM initiating SGLT2is versus DPP-4is, and SGLT2is versus GLP-1RAs. The propensity score was based on the following variables:

- **Demographics**: age, sex, socioeconomic status.
- Duration of T2DM, metformin use (purchased more than three times in the year preceding the index date) smoking status (current/past/never).
- **Laboratory tests**: creatinine, hemoglobin at the index date.
- **Comorbidities**: obesity, hypertension, MI, stroke, heart failure, atrial fibrillation (AF), venous thromboembolism (VTE), ischemic heart diseases (IHD), peripheral vascular disease (PVD), chronic kidney disease (CKD), obstructive lung diseases, obstructive sleep apnea (OSA), liver diseases and malignancy.
- **Health care utilization**: hospitalizations in the year prior to the index date.

For the propensity score calculation, we used a multivariable logistic regression model with the SGLT2i, DPP-4i, or GLP-1RA treatment group as the dependent variable and the variables listed above as the explanatory variables. We used the nearest neighbor method with a caliper size of 0.2 standard deviations of the propensity score in a 1:1 ratio. Standardized mean difference (SMD) was estimated to assess covariate balance before and after matching. Statistically significant standardized differences were considered at a cutoff of 0.1 or higher.

© 2025 Lewis M et al. *JAMA Network Open*.

eFigure 1. Flowchart of Patients Included in the SGLT2i vs DPP-4i Cohort

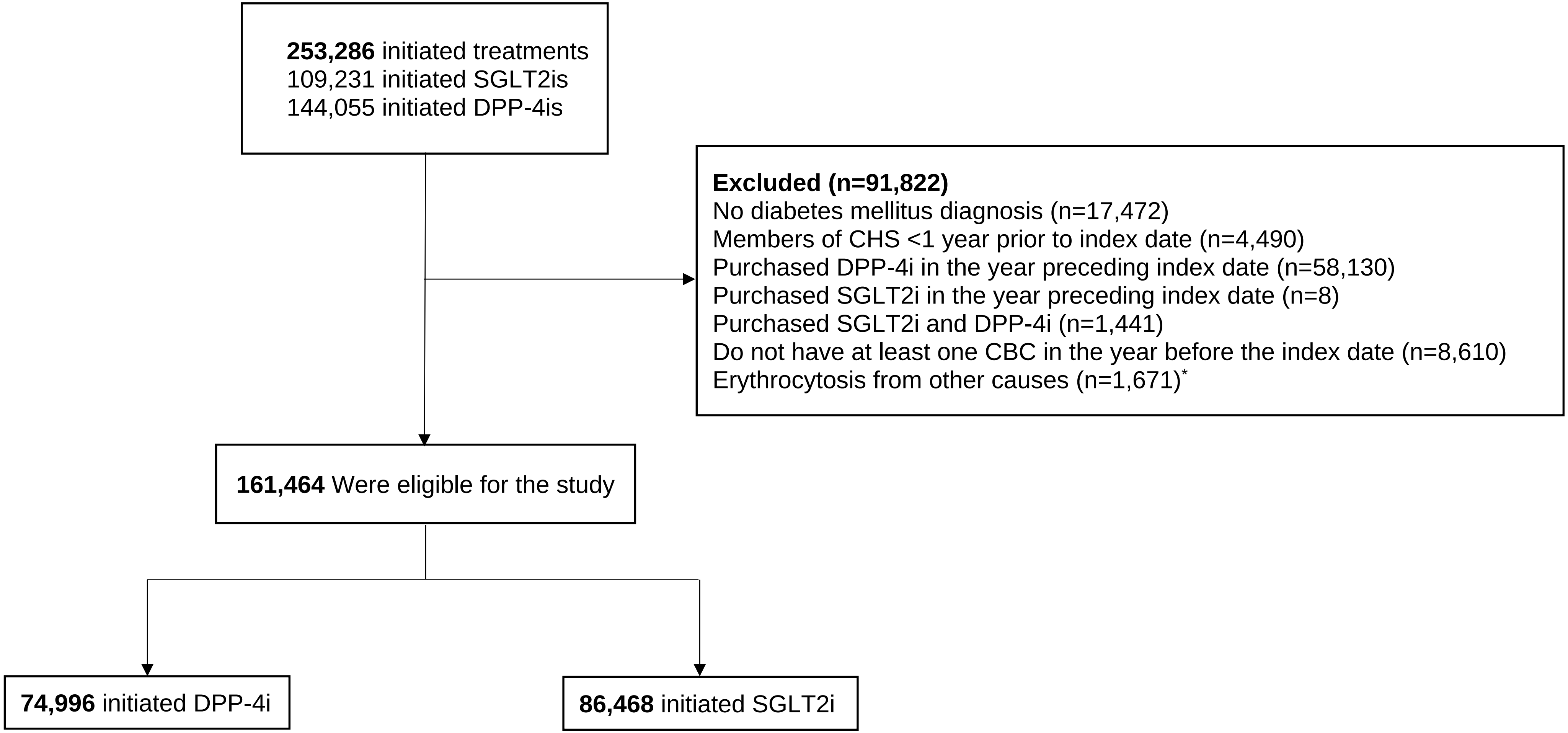

CHS – Clalit Health Services; DPP-4i – dipeptidyl peptidase-4 inhibitor; SGLT2i – sodium–glucose cotransporter-2 inhibitor; CBC – complete blood count.
* Diagnoses of familial polycythemia or myeloproliferative disorders at any time point or secondary erythrocytosis in the year prior to the index date.

© 2025 Lewis M et al. *JAMA Network Open*.

eFigure 2. – Flowchart of Patients Included in the SGLT2i vs. GLP-1RA Cohort

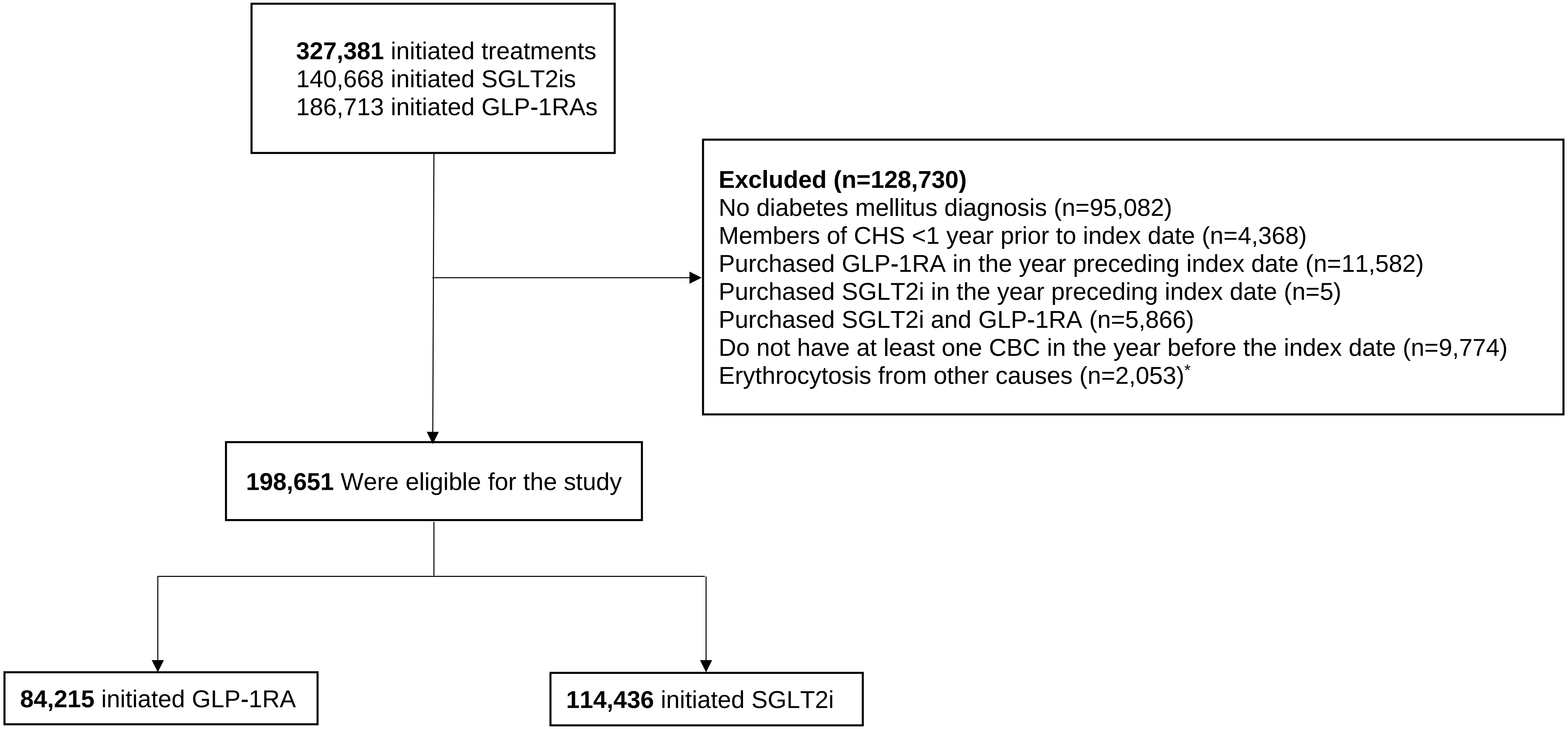

CHS – Clalit Health Services; GLP-1RA – glucagon-like peptide 1 receptor agonist; SGLT2i – sodium–glucose cotransporter-2 inhibitor; CBC – complete blood count.
* Diagnoses of familial polycythemia or myeloproliferative disorders at any time point or secondary erythrocytosis in the year prior to the index date.

© 2025 Lewis M et al. *JAMA Network Open*.

eFigure 3. Hemoglobin (g/dL) and Hematocrit (%) Levels Over Time Among Patients With Type 2 Diabetes Initiating SGLT2is vs DPP-4is After Propensity Score Matching, Divided by Sex

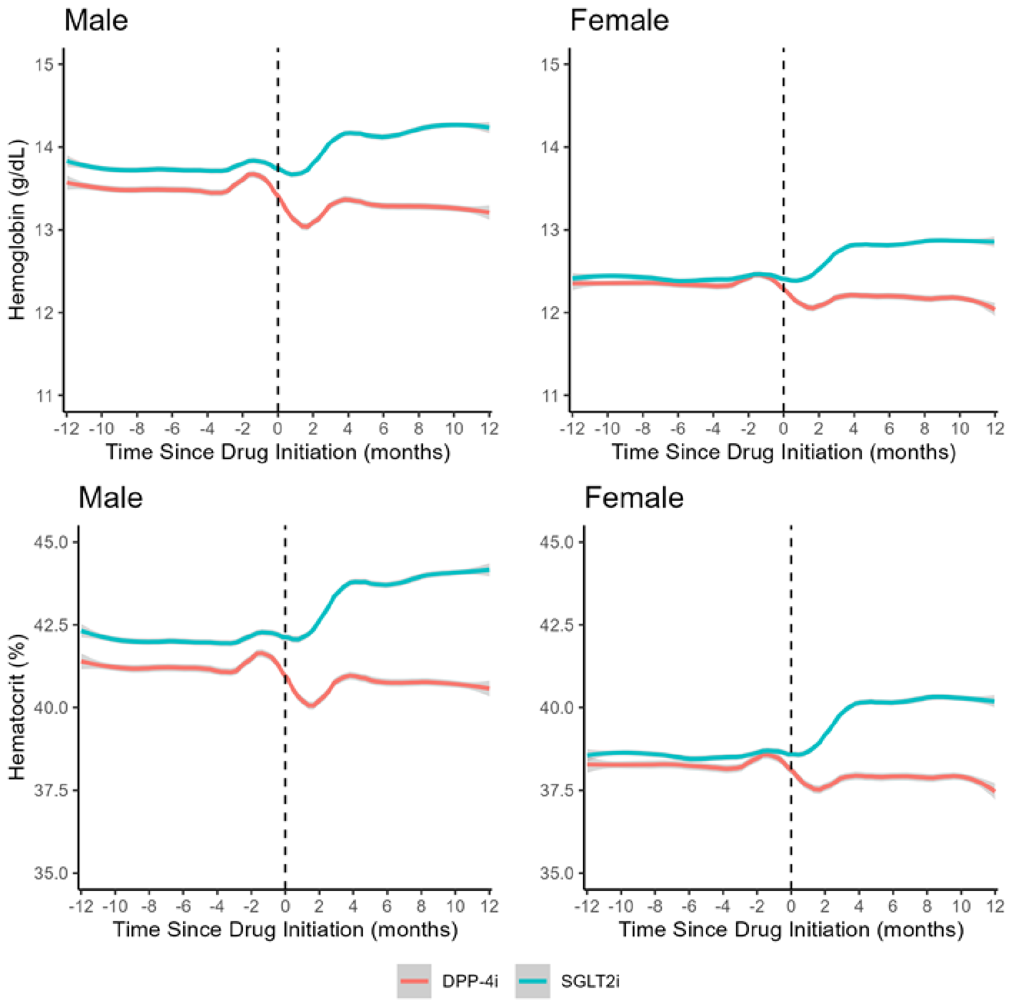

Lines were smoothed using the LOWESS method. The shaded areas around the curves indicate 95% CIs.

DPP-4i – dipeptidyl peptidase-4 inhibitor; SGLT2i – sodium–glucose cotransporter-2 inhibitor.

© 2025 Lewis M et al. *JAMA Network Open*.

eFigure 4. Hemoglobin (g/dL) and Hematocrit (%) Levels Over Time Among Patients With Type 2 Diabetes Initiating SGLT2is vs GLP-1RAs After Propensity Score Matching, Divided by Sex

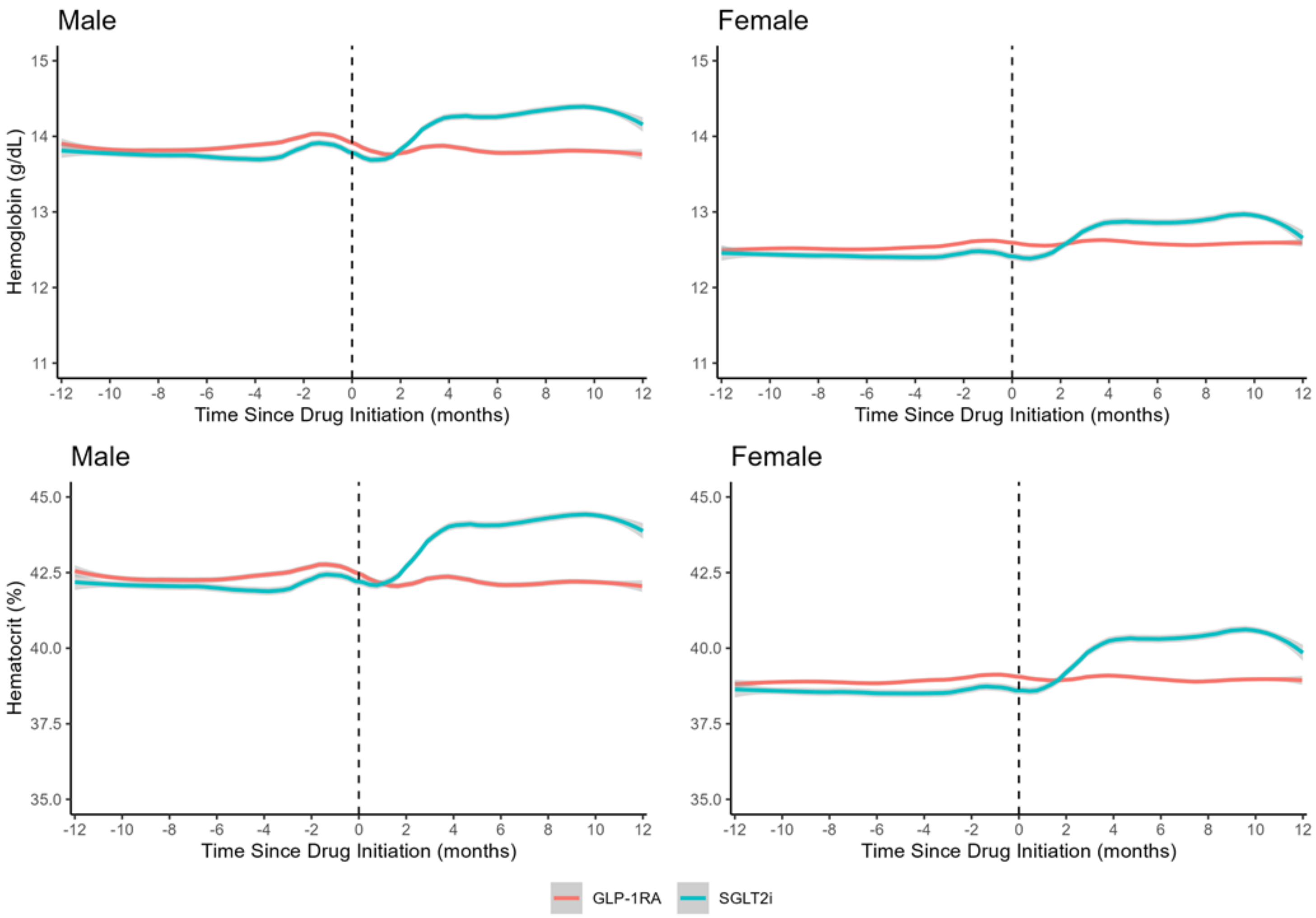

Lines were smoothed using the LOWESS method. The shaded areas around the curves indicate 95% CIs.

GLP-1RA – glucagon-like peptide 1 receptor agonist; SGLT2i – sodium–glucose cotransporter-2 inhibitor.

© 2025 Lewis M et al. *JAMA Network Open*.

eFigure 5. Percentage of Erythrocytosis (Lenient Criteria) Over Time Among Patients With Type 2 Diabetes Initiating SGLT2is vs DPP-4is After Propensity Matching Score Matching, Divided by Sex

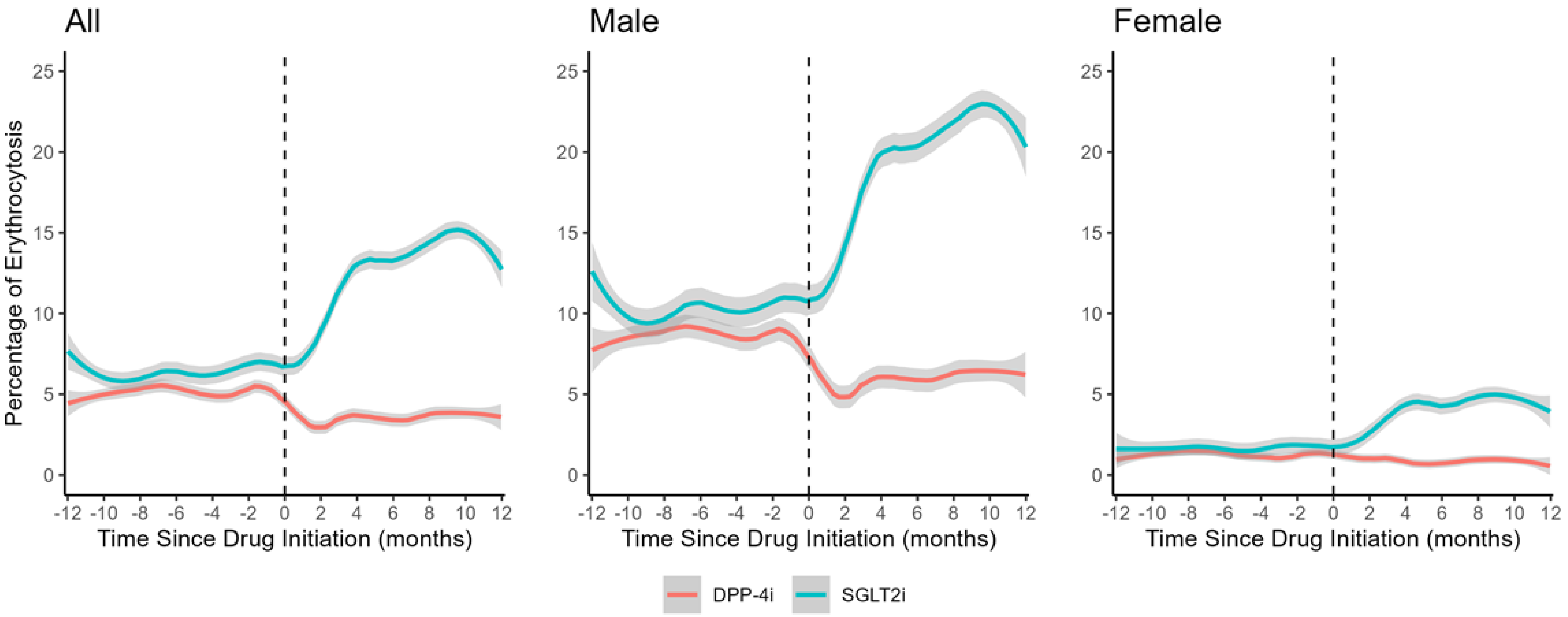

Lines were smoothed using the LOWESS method. The shaded areas around the curves indicate 95% CIs.

DPP-4i – dipeptidyl peptidase-4 inhibitor; SGLT2i – sodium–glucose cotransporter-2 inhibitor.

© 2025 Lewis M et al. *JAMA Network Open*.

eFigure 6. Percentage of Erythrocytosis (Lenient Criteria) Over Time Among Patients With Type 2 Diabetes Initiating SGLT2is vs GLP1-RAs After Propensity Matching Score Matching, Divided by Sex

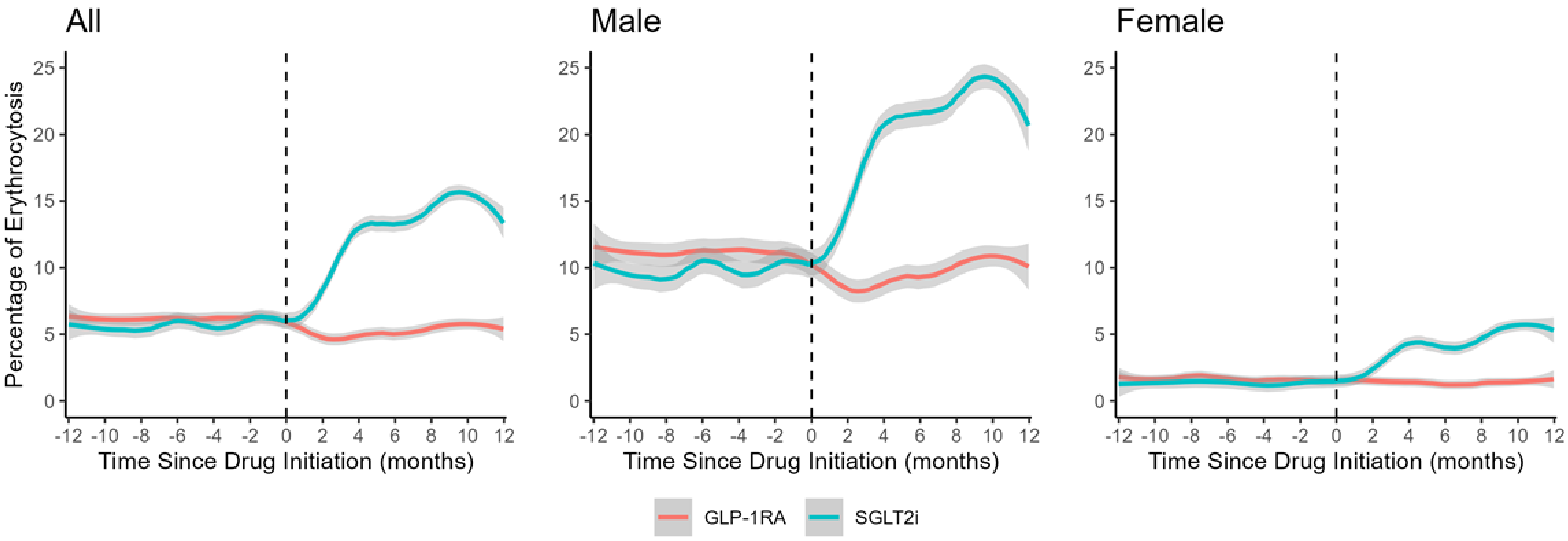

Lines were smoothed using the LOWESS method. The shaded areas around the curves indicate 95% CIs.

GLP-1RA – glucagon-like peptide 1 receptor agonist; SGLT2i – sodium–glucose cotransporter-2 inhibitor.

© 2025 Lewis M et al. *JAMA Network Open*.

eFigure 7. Hemoglobin (g/dL) and Hematocrit (%) Levels Over Time Among Patients With Type 2 Diabetes Initiating SGLT2is (Unmatched Cohort, Cohort 3), Divided by Sex

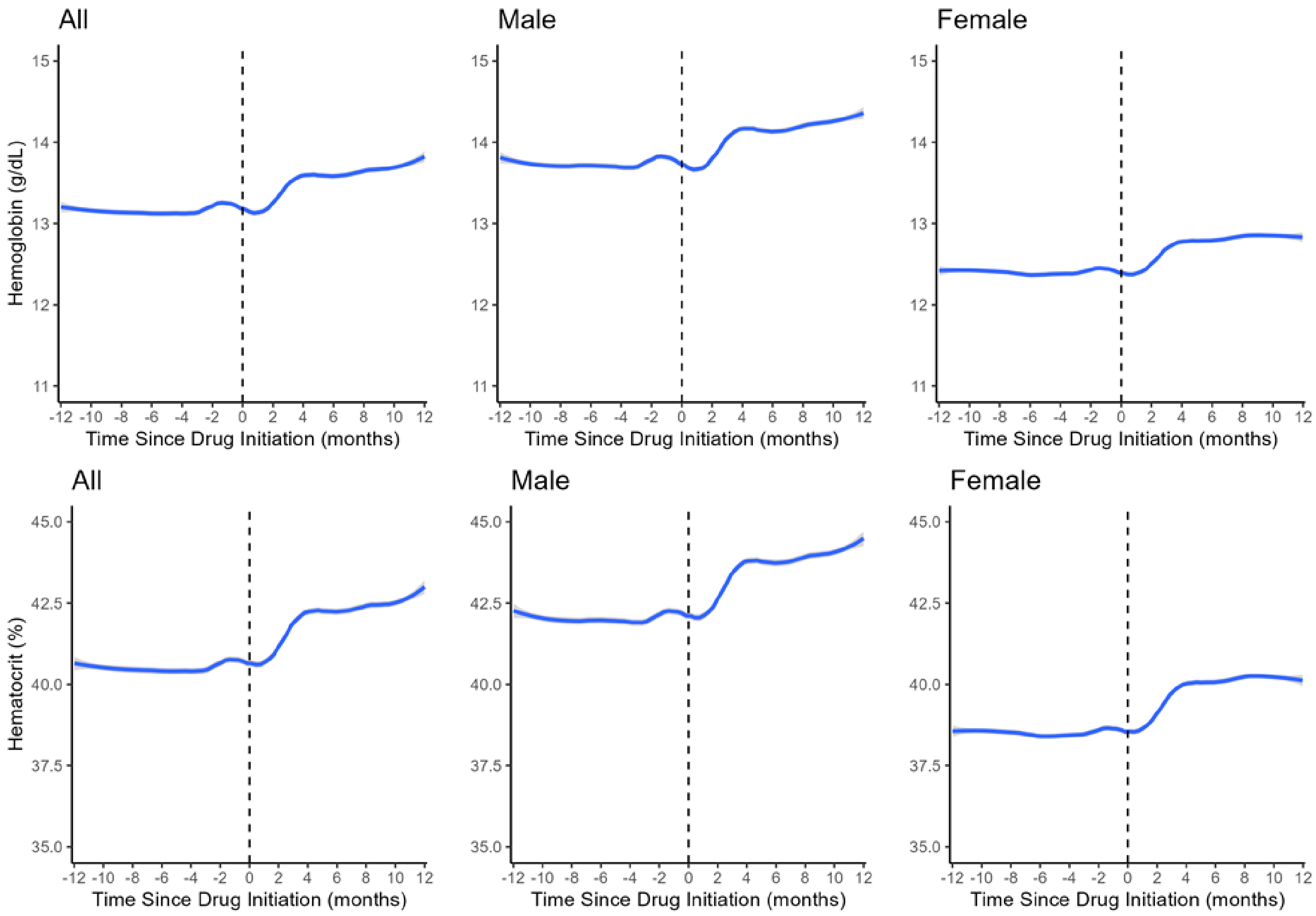

Lines were smoothed using the LOWESS method. The shaded areas around the curves indicate 95% CIs.
SGLT2i – sodium–glucose cotransporter-2 inhibitor.

© 2025 Lewis M et al. *JAMA Network Open*.

eFigure 8. Hemoglobin (g/dL) and Hematocrit (%) Levels Over Time Among Patients With Type 2 Diabetes Initiating SGLT2is (Unmatched Cohort, Cohort 3), Divided by Smoking Status

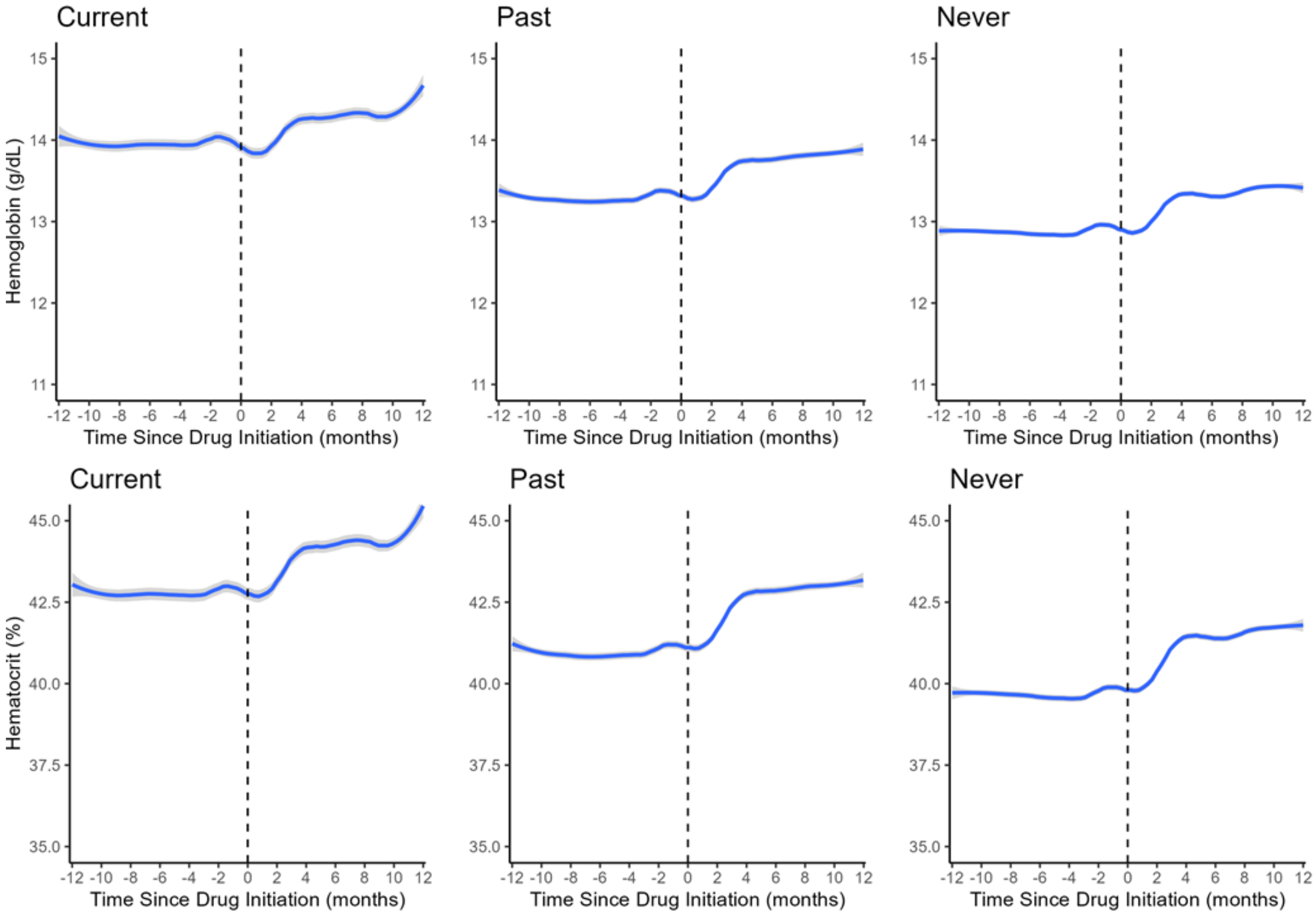

Lines were smoothed using the LOWESS method. The shaded areas around the curves indicate 95% CIs.
SGLT2i – sodium–glucose cotransporter-2 inhibitor.

© 2025 Lewis M et al. *JAMA Network Open*.

eFigure 9. Hemoglobin (g/dL) and Hematocrit (%) Levels Over Time Among Patients With Type 2 Diabetes Initiating SGLT2is (Unmatched Cohort, Cohort 3), Divided by SGLT2i Type

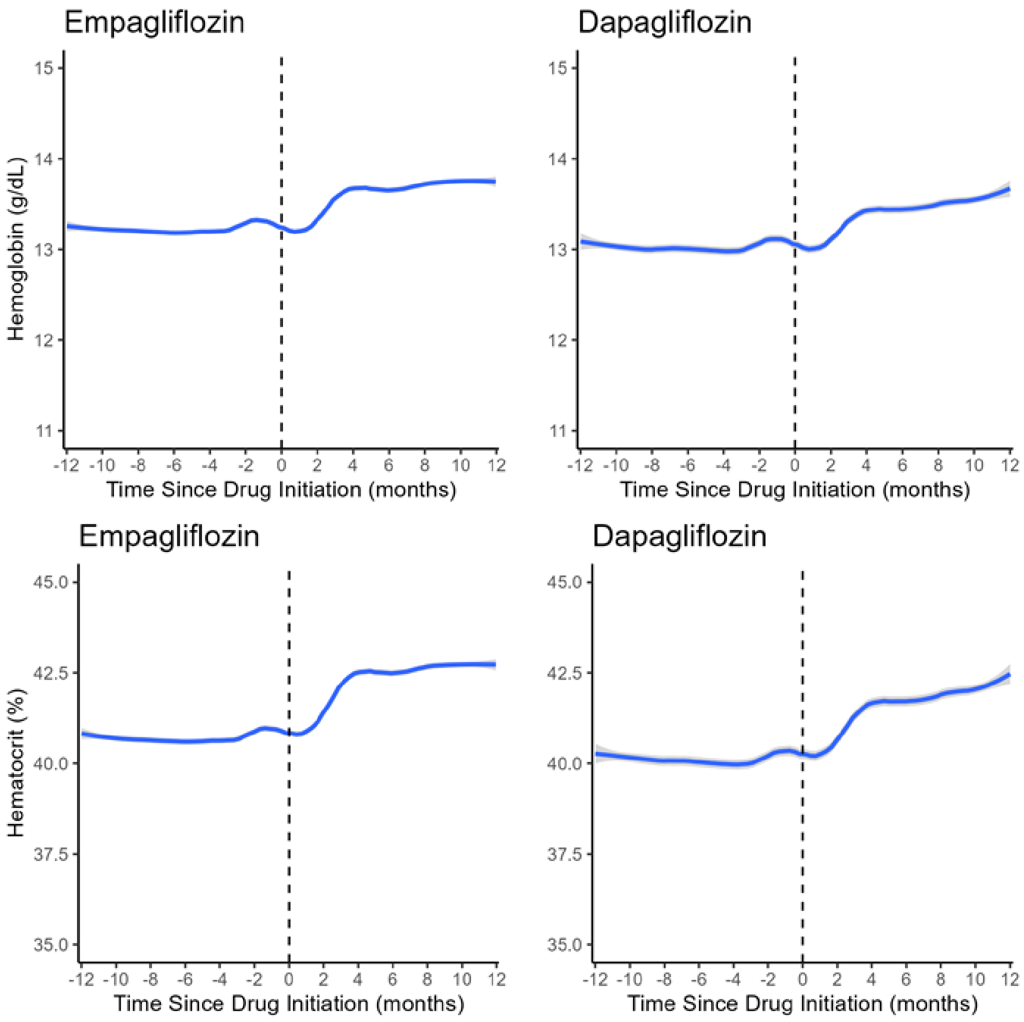

Lines were smoothed using the LOWESS method. The shaded areas around the curves indicate 95% CIs.
SGLT2i – sodium–glucose cotransporter-2 inhibitor.

© 2025 Lewis M et al. *JAMA Network Open*.

eFigure 10. Kaplan-Meier Curves for MI (A), VTE (B), and Stroke (C) in SGLT2i Initiators With and Without New-Onset Erythrocytosis (Lenient Criteria)

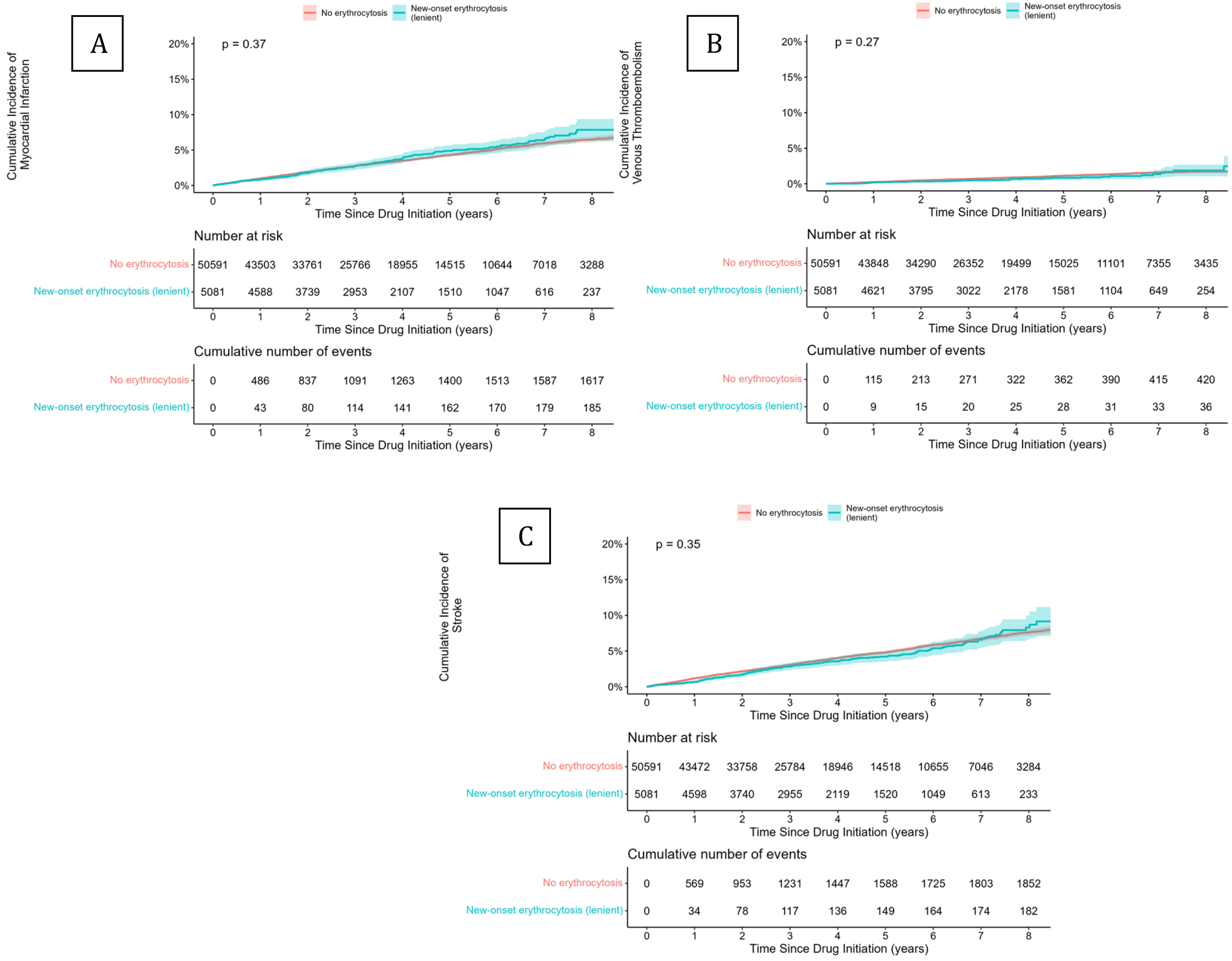

The shaded areas around the curves indicate 95% CIs. SGLT2i – sodium–glucose cotransporter-2 inhibitor; MI – myocardial infarction; VTE – venous thromboembolism.

© 2025 Lewis M et al. *JAMA Network Open*.

eFigure 11. Kaplan-Meier Curves for MI (A), VTE (B), and Stroke (C) in SGLT2i Initiators With and Without New-Onset Erythrocytosis (Strict Criteria)

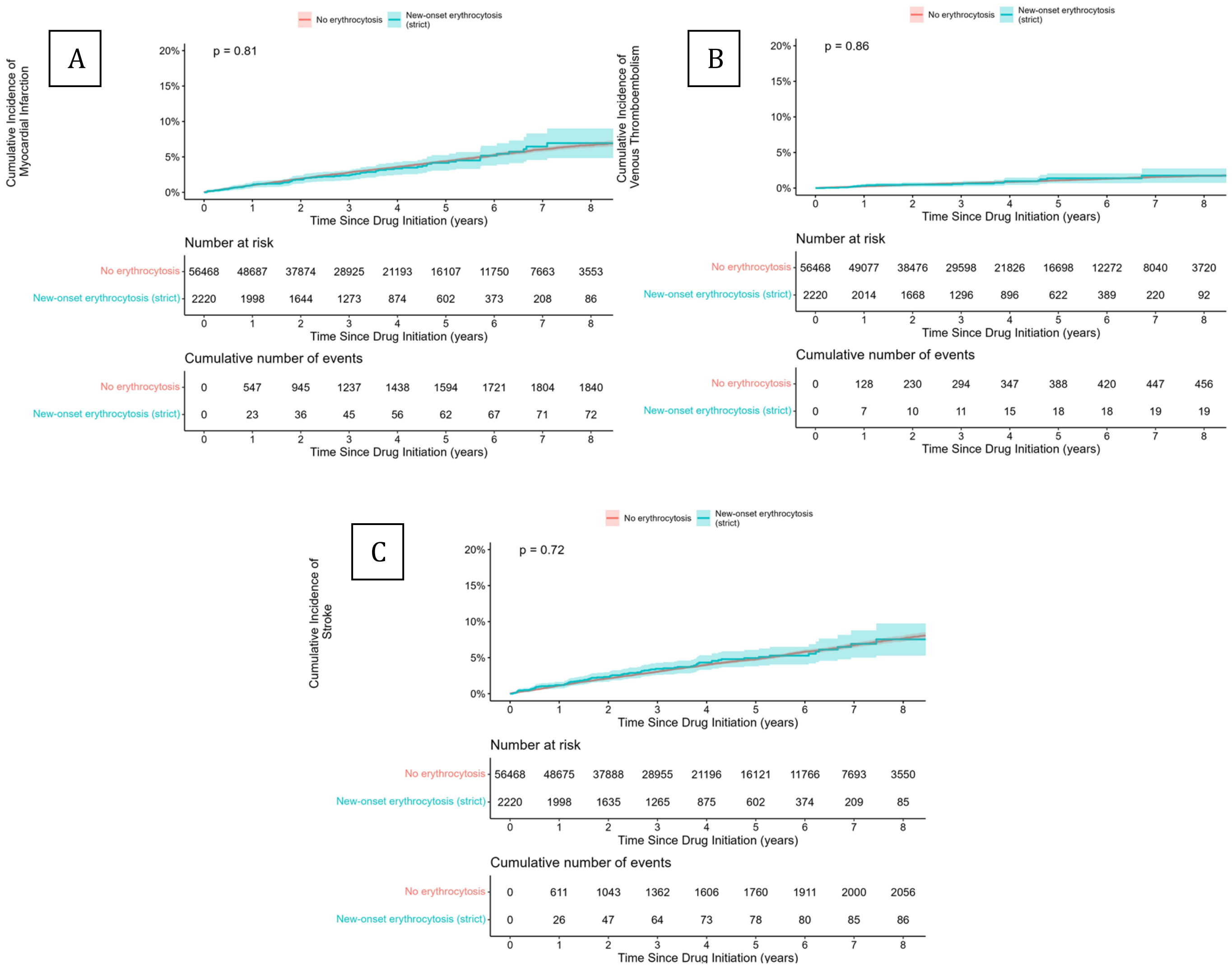

The shaded areas around the curves indicate 95% CIs. SGLT2i – sodium–glucose cotransporter-2 inhibitor; MI – myocardial infarction; VTE – venous thromboembolism.

© 2025 Lewis M et al. *JAMA Network Open*.

eTable 1. Baseline Characteristics of Patients With Type 2 Diabetes Initiating SGLT2is vs DPP-4is, Before and After 1:1 Propensity Score Matching

	Unmatched			Matched		
**Characteristic**	**DPP-4i**, N = 74,996	**SGLT2i**, N = 86,468	**SMD (95% CI)**	**DPP-4i**, N = 68,776	**SGLT2i**, N = 68,776	**SMD (95% CI)**
Age, Mean ± SD	65.26 ± 13.04	64.50 ± 12.04	0.06 (0.05 to 0.07)	64.73 ± 13.08	64.55 ± 12.03	0.01 (0.00 to 0.02)
Male sex, n (%)	39,010 (52.0%)	50,745 (58.7%)	0.13 (0.12 to 0.14)	36,790 (53.5%)	38,053 (55.3%)	0.04 (0.03 to 0.05)
**Socioeconomic status, n (%)**			0.23 (0.22 to 0.24)			0.06 (0.05 to 0.07)
Very High	5,051 (6.7%)	5,212 (6.0%)		4,477 (6.5%)	4,370 (6.4%)	
High	17,172 (22.9%)	17,747 (20.5%)		15,365 (22.3%)	15,092 (21.9%)	
Medium	26,306 (35.1%)	26,757 (30.9%)		23,552 (34.2%)	22,683 (33.0%)	
Low	20,495 (27.3%)	24,748 (28.6%)		19,470 (28.3%)	19,656 (28.6%)	
Very Low	2,122 (2.8%)	6,229 (7.2%)		2,122 (3.1%)	2,771 (4.0%)	
Missing data	3,850 (5.1%)	5,775 (6.7%)		3,790 (5.5%)	4,204 (6.1%)	
**Smoking, n (%)**			0.12 (0.11 to 0.13)			0.03 (0.02 to 0.04)
Never	47,022 (62.7%)	49,265 (57.0%)		42,326 (61.5%)	41,335 (60.1%)	
Past	15,937 (21.3%)	21,630 (25.0%)		15,166 (22.1%)	15,915 (23.1%)	
Current	12,037 (16.1%)	15,573 (18.0%)		11,284 (16.4%)	11,526 (16.8%)	
**Comorbidities, n (%)**						
Obesity	44,752 (59.7%)	61,071 (70.6%)	-0.23 (-0.24 to -0.22)	43,913 (63.8%)	46,418 (67.5%)	-0.08 (-0.09 to -0.07)
Hypertension	51,272 (68.4%)	64,043 (74.1%)	-0.13 (-0.14 to -0.12)	47,661 (69.3%)	49,084 (71.4%)	-0.05 (-0.06 to -0.03)
Myocardial infarction	6,451 (8.6%)	16,803 (19.4%)	-0.32 (-0.33 to -0.31)	6,447 (9.4%)	8,258 (12.0%)	-0.09 (-0.10 to -0.07)
Stroke	6,992 (9.3%)	9,362 (10.8%)	-0.05 (-0.06 to -0.04)	6,462 (9.4%)	6,803 (9.9%)	-0.02 (-0.03 to -0.01)
Ischemic heart disease	17,956 (23.9%)	34,090 (39.4%)	-0.34 (-0.35 to -0.33)	17,762 (25.8%)	20,800 (30.2%)	-0.10 (-0.11 to -0.09)
Heart failure	7,190 (9.6%)	16,084 (18.6%)	-0.26 (-0.27 to -0.25)	7,128 (10.4%)	8,812 (12.8%)	-0.08 (-0.09 to -0.07)
Venous thromboembolism	3,261 (4.3%)	4,748 (5.5%)	-0.05 (-0.06 to -0.04)	3,118 (4.5%)	3,367 (4.9%)	-0.02 (-0.03 to -0.01)
Atrial fibrillation	7,004 (9.3%)	11,101 (12.8%)	-0.11 (-0.12 to -0.10)	6,660 (9.7%)	7,424 (10.8%)	-0.04 (-0.05 to -0.03)
Chronic kidney disease	12,447 (16.6%)	13,601 (15.7%)	0.02 (0.01 to 0.03)	10,999 (16.0%)	10,395 (15.1%)	0.02 (0.01 to 0.03)
Peripheral vascular disease	5,863 (7.8%)	7,445 (8.6%)	-0.03 (-0.04 to -0.02)	5,276 (7.7%)	5,487 (8.0%)	-0.01 (-0.02 to 0.00)
Obstructive lung diseases	10,591 (14.1%)	14,006 (16.2%)	-0.06 (-0.07 to -0.05)	9,946 (14.5%)	10,385 (15.1%)	-0.02 (-0.03 to -0.01)
Obstructive sleep apnea	845 (1.1%)	1,832 (2.1%)	-0.08 (-0.09 to -0.07)	840 (1.2%)	993 (1.4%)	-0.02 (-0.03 to -0.01)
Liver diseases	13,126 (17.5%)	18,004 (20.8%)	-0.08 (-0.09 to -0.07)	12,735 (18.5%)	13,700 (19.9%)	-0.04 (-0.05 to -0.03)
Malignancy	10,705 (14.3%)	11,422 (13.2%)	0.03 (0.02 to 0.04)	9,468 (13.8%)	9,345 (13.6%)	0.01 (-0.01 to 0.02)
**Blood tests** *						
HbA1c, %, Mean ± SD	8.14 ± 1.67	8.10 ± 1.75	0.02 (0.01 to 0.03)	8.16 ± 1.67	8.08 ± 1.72	0.04 (0.03 to 0.06)
Creatinine, mg/dL, Mean ± SD	0.93 ± 0.55	0.89 ± 0.30	0.10 (0.10 to 0.11)	0.92 ± 0.55	0.88 ± 0.30	0.09 (0.08 to 0.10)
Estimated glomerular filtration rate (mL/min/1.73m2), Mean ± SD	81.81 ± 24.40	83.49 ± 21.59	-0.07 (-0.08 to -0.06)	83.02 ± 24.16	83.19 ± 21.49	-0.01 (-0.02 to 0.00)

© 2025 Lewis M et al. *JAMA Network Open*.

Hemoglobin (g/dL), Mean ± SD	13.40 ± 1.66	13.53 ± 1.66	-0.08 (-0.09 to -0.07)	13.45 ± 1.65	13.48 ± 1.64	-0.02 (-0.03 to -0.01)
Days to first CBC after index date, Median (IQR)	106.41 (54.37, 182)	100.38 (50.31, 171)	0.08 (0.06 to 0.09)	107.38 (55.32, 183)	103.30 (52.39, 173)	0.06 (0.05 to 0.07)
Hospitalizations, Mean ± SD†	0.36 ± 0.91	0.48 ± 1.03	-0.13 (-0.14 to -0.12)	0.37 ± 0.92	0.40 ± 0.92	-0.03 (-0.04 to -0.02)
Duration of diabetes, years, Mean ± SD	10.35 ± 7.87	11.06 ± 7.57	-0.09 (-0.10 to -0.08)	10.45 ± 7.96	10.71 ± 7.37	-0.03 (-0.04 to -0.02)
Metformin use, n (%)‡	66,161 (88.2%)	71,750 (83.0%)	0.15 (0.14 to 0.16)	60,229 (87.6%)	59,326 (86.3%)	0.04 (0.03 to 0.05)

SMD – standardized mean difference; CI – confidence interval; DPP-4i – dipeptidyl peptidase-4 inhibitor; SGLT2i – sodium-glucose cotransporter-2 inhibitors; CBC – complete blood count.
* Most recent value in the year preceding the index date.
† Frequency during the previous year.
‡ Defined as those purchased metformin at least three times in the year preceding the index date.

© 2025 Lewis M et al. *JAMA Network Open*.

eTable 2. Baseline Characteristics of Patients With Type 2 Diabetes Initiating SGLT2is vs GLP-1RAs, Before and After 1:1 Propensity Score Matching

	Unmatched			Matched		
**Characteristic**	**GLP-1RA**, N = 84,215	**SGLT2i**, N = 114,436	**SMD (95% CI)**	**GLP-1RA**, N = 65,756	**SGLT2i**, N = 65,756	**SMD (95% CI)**
Age, Mean ± SD	59.90 ± 12.79	66.18 ± 11.61	-0.51 (-0.52 to -0.50)	62.77 ± 11.56	63.73 ± 11.87	-0.08 (-0.09 to -0.07)
Male sex, n (%)	36,220 (43.0%)	69,759 (61.0%)	0.37 (0.36 to 0.37)	31,925 (48.6%)	34,195 (52.0%)	0.07 (0.06 to 0.08)
**Socioeconomic status, n (%)**			0.29 (0.28 to 0.29)			0.05 (0.04 to 0.06)
Very High	4,556 (5.4%)	8,317 (7.3%)		3,950 (6.0%)	4,167 (6.3%)	
High	15,702 (18.6%)	27,531 (24.1%)		13,514 (20.6%)	14,166 (21.5%)	
Medium	24,580 (29.2%)	38,887 (34.0%)		20,666 (31.4%)	21,256 (32.3%)	
Low	26,053 (30.9%)	29,034 (25.4%)		19,744 (30.0%)	18,808 (28.6%)	
Very Low	7,375 (8.8%)	4,411 (3.9%)		3,637 (5.5%)	3,388 (5.2%)	
Missing data	5,949 (7.1%)	6,256 (5.5%)		4,245 (6.5%)	3,971 (6.0%)	
**Smoking, n (%)**			0.11 (0.10 to 0.12)			0.02 (0.01 to 0.03)
Never	52,658 (62.5%)	65,710 (57.4%)		39,918 (60.7%)	39,314 (59.8%)	
Past	17,935 (21.3%)	29,232 (25.5%)		15,153 (23.0%)	15,601 (23.7%)	
Current	13,622 (16.2%)	19,494 (17.0%)		10,685 (16.2%)	10,841 (16.5%)	
**Comorbidities**						
Obesity	74,331 (88.3%)	67,944 (59.4%)	0.70 (0.69 to 0.70)	55,892 (85.0%)	52,574 (80.0%)	0.13 (0.12 to 0.14)
Hypertension	54,665 (64.9%)	84,360 (73.7%)	-0.19 (-0.20 to -0.18)	46,450 (70.6%)	47,177 (71.7%)	-0.02 (-0.04 to -0.01)
Myocardial infarction	5,349 (6.4%)	20,619 (18.0%)	-0.36 (-0.37 to -0.35)	5,326 (8.1%)	7,241 (11.0%)	-0.10 (-0.11 to -0.09)
Stroke	6,635 (7.9%)	11,666 (10.2%)	-0.08 (-0.09 to -0.07)	5,787 (8.8%)	6,101 (9.3%)	-0.02 (-0.03 to -0.01)
Ischemic heart disease	15,294 (18.2%)	43,106 (37.7%)	-0.45 (-0.45 to -0.44)	14,948 (22.7%)	17,865 (27.2%)	-0.10 (-0.11 to -0.09)
Heart failure	6,276 (7.5%)	18,438 (16.1%)	-0.27 (-0.28 to -0.26)	6,091 (9.3%)	7,769 (11.8%)	-0.08 (-0.09 to -0.07)
Venous thromboembolism	4,621 (5.5%)	5,288 (4.6%)	0.04 (0.03 to 0.05)	3,502 (5.3%)	3,413 (5.2%)	0.01 (0.00 to 0.02)
Atrial fibrillation	5,713 (6.8%)	13,886 (12.1%)	-0.18 (-0.19 to -0.17)	5,443 (8.3%)	6,403 (9.7%)	-0.05 (-0.06 to -0.04)
Chronic kidney disease	9,781 (11.6%)	18,081 (15.8%)	-0.12 (-0.13 to -0.11)	8,935 (13.6%)	9,150 (13.9%)	-0.01 (-0.02 to 0.00)
Peripheral vascular disease	4,789 (5.7%)	9,777 (8.5%)	-0.11 (-0.12 to -0.10)	4,301 (6.5%)	4,760 (7.2%)	-0.03 (-0.04 to -0.02)
Obstructive lung diseases	14,150 (16.8%)	16,242 (14.2%)	0.07 (0.06 to 0.08)	10,488 (15.9%)	10,278 (15.6%)	0.01 (0.00 to 0.02)
Obstructive sleep apnea	1,643 (2.0%)	1,734 (1.5%)	0.03 (0.02 to 0.04)	1,263 (1.9%)	1,220 (1.9%)	0.00 (-0.01 to 0.02)
Liver diseases	19,656 (23.3%)	22,499 (19.7%)	0.09 (0.08 to 0.10)	15,236 (23.2%)	14,697 (22.4%)	0.02 (0.01 to 0.03)
Malignancy	9,277 (11.0%)	16,415 (14.3%)	-0.10 (-0.11 to -0.09)	8,089 (12.3%)	8,495 (12.9%)	-0.02 (-0.03 to -0.01)
**Blood tests** *						
HbA1c, %, Mean ± SD	8.21 ± 1.90	8.03 ± 1.63	0.10 (0.09 to 0.11)	8.23 ± 1.84	8.10 ± 1.67	0.07 (0.06 to 0.08)
Creatinine, mg/dL, Mean ± SD	0.83 ± 0.40	0.90 ± 0.31	-0.18 (-0.19 to -0.18)	0.87 ± 0.41	0.87 ± 0.30	0.00 (-0.01 to 0.01)

© 2025 Lewis M et al. *JAMA Network Open*.

Estimated glomerular filtration rate (mL/min/1.73m2), Mean ± SD	89.07 ± 22.69	81.76 ± 21.08	0.33 (0.32 to 0.34)	85.64 ± 22.32	84.35 ± 21.45	0.06 (0.05 to 0.07)
Hemoglobin (g/dL), Mean ± SD	13.45 ± 1.60	13.49 ± 1.61	-0.02 (-0.03 to -0.01)	13.48 ± 1.59	13.47 ± 1.62	0.01 (-0.01 to 0.02)
Days to first CBC after index date, Median (IQR)	105.41 (58.31, 177)	102.31 (51.35, 174)	0.05 (0.04 to 0.06)	104.38 (56.44, 174)	105.32 (54.40, 178)	-0.01 (-0.02 to 0.00)
Hospitalizations, Mean ± SD†	0.28 ± 0.76	0.43 ± 0.95	-0.17 (-0.18 to -0.16)	0.31 ± 0.81	0.35 ± 0.86	-0.05 (-0.06 to -0.04)
Duration of diabetes, years, Mean ± SD	9.95 ± 7.21	12.08 ± 7.92	-0.28 (-0.29 to -0.27)	10.74 ± 7.32	11.14 ± 7.48	-0.05 (-0.07 to -0.04)
Metformin use, n (%)‡	43,849 (52.1%)	78,792 (68.9%)	-0.35 (-0.36 to -0.34)	40,976 (62.3%)	41,905 (63.7%)	-0.03 (-0.04 to -0.02)

SMD – standardized mean difference; CI – confidence interval; GLP-1RA – glucagon-like peptide-1 receptor agonist; SGLT2i – sodium-glucose cotransporter-2 inhibitor; CBC – complete blood count.
* Most recent value in the year preceding the index date.
† Frequency during the previous year.
‡ Defined as those purchased metformin at least three times in the year preceding the index date.

© 2025 Lewis M et al. *JAMA Network Open*.

eTable 3. Erythrocytosis Rates Among Patients With Type 2 Diabetes Before and After Initiating SGLT2is (Cohort 3)

Group	**Before** N = 86,467	**After** N = 59,852	Difference (95% CI)	p-value
**All patients, n (%)**				
Erythrocytosis (lenient criteria)	6,274 (7.3%)	7,700 (12.9%)	5.6% (5.3% to 5.9%)	<0.001
Erythrocytosis (strict criteria)	1,643 (1.9%)	2,699 (4.5%)	2.6% (2.4% to 2.8%)	<0.001
**Males, n (%)**				
Erythrocytosis (lenient criteria)	5,661 (11.2%)	6,738 (19.2%)	8.1% (7.6% to 8.6%)	<0.001
Erythrocytosis (strict criteria)	1,068 (2.1%)	1,782 (5.1%)	3.0% (2.7% to 3.2%)	<0.001
**Females, n (%)**				
Erythrocytosis (lenient criteria)	613 (1.7%)	962 (3.9%)	2.2% (1.9% to 2.4%)	<0.001
Erythrocytosis (strict criteria)	575 (1.6%)	917 (3.7%)	2.1% (1.8% to 2.4%)	<0.001

SGLT2is – sodium–glucose cotransporter-2 inhibitors.

© 2025 Lewis M et al. *JAMA Network Open*.

eTable 4. Erythrocytosis Rates Among Patients With Type 2 Diabetes Before and After Initiating SGLT2is (Cohort 3), Stratified by 3-Month Quartiles in the Year After Treatment Initiation

Group	Before<br>N = 86,467	0-3 months<br>N = 31,606	3-6 months<br>N = 30,133	6-9 months<br>N = 22,728	9-12 months<br>N = 19,451	p-value
**All patients, n (%)**						
Erythrocytosis (lenient criteria)	6,274 (7.3%)	2,254 (7.1%)	3,337 (11.1%)	2,620 (11.5%)	2,353 (12.1%)	<0.001
Erythrocytosis (strict criteria)	1,643 (1.9%)	683 (2.2%)	1,137 (3.8%)	911 (4.0%)	832 (4.3%)	<0.001
**Males, n (%)**						
Erythrocytosis (lenient criteria)	5,661 (11.2%)	1,999 (11.0%)	2,921 (16.5%)	2,300 (17.2%)	2,080 (18.0%)	<0.001
Erythrocytosis (strict criteria)	1,068 (2.1%)	445 (2.4%)	737 (4.2%)	607 (4.5%)	570 (4.9%)	<0.001
**Females, n (%)**						
Erythrocytosis (lenient criteria)	613 (1.7%)	255 (1.9%)	416 (3.3%)	320 (3.4%)	273 (3.5%)	<0.001
Erythrocytosis (strict criteria)	575 (1.6%)	238 (1.8%)	400 (3.2%)	304 (3.2%)	262 (3.3%)	<0.001

SGLT2is – sodium–glucose cotransporter-2 inhibitors.

© 2025 Lewis M et al. *JAMA Network Open*.

eTable 5. Erythrocytosis Rates Among Patients With Type 2 Diabetes, Before and After Initiating SGLT2is vs DPP-4is, After Propensity Score Matching, Stratified by 3-Month Quartiles in the Year After Treatment Initiation

	**DPP-4is**						**SGLT2is**					
**Group**	**Before** N = 68,721	**0-3 months** N = 22,522	**3-6 months** N = 21,443	**6-9 months** N = 16,806	**9-12 months** N = 14,970	**p-value**	**Before** N = 68,775	**0-3 months** N = 24,353	**3-6 months** N = 23,712	**6-9 months** N = 17,655	**9-12 months** N = 15,098	**p-value**
**All patients, n (%)**												
Erythrocytosis (lenient criteria)	3,863 (5.6%)	565 (2.5%)	548 (2.6%)	443 (2.6%)	384 (2.6%)	<0.001	4,601 (6.7%)	1,642 (6.7%)	2,480 (10.5%)	1,920 (10.9%)	1,720 (11.4%)	<0.001
Erythrocytosis (strict criteria)	945 (1.4%)	161 (0.7%)	141 (0.7%)	112 (0.7%)	90 (0.6%)	<0.001	1,206 (1.8%)	519 (2.1%)	849 (3.6%)	681 (3.9%)	609 (4.0%)	<0.001
**Males, n (%)**												
Erythrocytosis (lenient criteria)	3,416 (9.3%)	480 (4.1%)	492 (4.4%)	394 (4.6%)	339 (4.3%)	<0.001	4,093 (10.8%)	1,429 (10.8%)	2,126 (16.2%)	1,654 (16.9%)	1,499 (17.8%)	<0.001
Erythrocytosis (strict criteria)	542 (1.5%)	81 (0.7%)	89 (0.8%)	66 (0.8%)	50 (0.6%)	<0.001	730 (1.9%)	318 (2.4%)	509 (3.9%)	427 (4.4%)	395 (4.7%)	<0.001
**Females, n (%)**												
Erythrocytosis (lenient criteria)	447 (1.4%)	85 (0.8%)	56 (0.6%)	49 (0.6%)	45 (0.6%)	<0.001	508 (1.7%)	213 (1.9%)	354 (3.3%)	266 (3.4%)	221 (3.3%)	<0.001
Erythrocytosis (strict criteria)	403 (1.3%)	80 (0.7%)	52 (0.5%)	46 (0.6%)	40 (0.6%)	<0.001	476 (1.5%)	201 (1.8%)	340 (3.2%)	254 (3.2%)	214 (3.2%)	<0.001

DPP-4is – dipeptidyl peptidase-4 inhibitors; SGLT2is – sodium–glucose cotransporter-2 inhibitors.

© 2025 Lewis M et al. *JAMA Network Open*.

eTable 6. Erythrocytosis Rates Among Patients With Type 2 Diabetes, Before and After Initiating SGLT2is vs GLP-1RAs, After Propensity Score Matching, Stratified by 3-Month Quartiles in the Year After Treatment Initiation

	**GLP-1RAs**						**SGLT2is**					
**Group**	**Before** N = 65,694	**0-3 months** N = 22,731	**3-6 months** N = 22,005	**6-9 months** N = 15,972	**9-12 months** N = 13,282	**p-value**	**Before** N = 65,756	**0-3 months** N = 21,899	**3-6 months** N = 20,689	**6-9 months** N = 15,645	**9-12 months** N = 13,223	**p-value**
**All patients, n (%)**												
Erythrocytosis (lenient criteria)	4,124 (6.3%)	948 (4.2%)	839 (3.8%)	598 (3.7%)	532 (4.0%)	<0.001	4,032 (6.1%)	1,382 (6.3%)	2,124 (10.3%)	1,710 (10.9%)	1,563 (11.8%)	<0.001
Erythrocytosis (strict criteria)	1,112 (1.7%)	270 (1.2%)	242 (1.1%)	165 (1.0%)	148 (1.1%)	<0.001	1,076 (1.6%)	432 (2.0%)	770 (3.7%)	596 (3.8%)	603 (4.6%)	<0.001
**Males, n (%)**												
Erythrocytosis (lenient criteria)	3,571 (11.2%)	804 (7.5%)	719 (6.9%)	524 (7.1%)	460 (7.6%)	<0.001	3,586 (10.5%)	1,189 (10.9%)	1,809 (16.8%)	1,463 (18.0%)	1,303 (18.9%)	<0.001
Erythrocytosis (strict criteria)	603 (1.9%)	134 (1.3%)	131 (1.3%)	98 (1.3%)	84 (1.4%)	<0.001	654 (1.9%)	248 (2.3%)	467 (4.3%)	357 (4.4%)	351 (5.1%)	<0.001
**Females, n (%)**												
Erythrocytosis (lenient criteria)	553 (1.6%)	144 (1.2%)	120 (1.0%)	74 (0.9%)	72 (1.0%)	<0.001	446 (1.4%)	193 (1.8%)	315 (3.2%)	247 (3.3%)	260 (4.1%)	<0.001
Erythrocytosis (strict criteria)	509 (1.5%)	136 (1.1%)	111 (1.0%)	67 (0.8%)	64 (0.9%)	<0.001	422 (1.3%)	184 (1.7%)	303 (3.1%)	239 (3.2%)	252 (4.0%)	<0.001

GLP-1RAs – glucagon-like peptide-1 receptor agonists; SGLT2is – sodium–glucose cotransporter-2 inhibitors.

© 2025 Lewis M et al. *JAMA Network Open*.

eTable 7. Hemoglobin (g/dL) and Hematocrit (%) Levels Among Patients With Type 2 Diabetes, Before and After Initiating SGLT2is vs DPP-4is, After Propensity Score Matching

		DPP-4is			SGLT2is		
**Group**	**Characteristic**	**Before** N = 44,958	**After** N = 44,958	**Difference (95% CI)**	**Before** N = 46,993	**After** N = 46,993	**Difference (95% CI)**
**All patients, n (%)**							
Hemoglobin (g/dL)	Mean ± SD	13.28 ± 1.61	13.09 ± 1.62	-0.19 (-0.20 to -0.18)	13.37 ± 1.59	13.72 ± 1.72	0.35 (0.34 to 0.36)
	Median (IQR)	13.30 (12.20, 14.40)	13.15 (12.03, 14.20)		13.40 (12.30, 14.50)	13.80 (12.60, 14.90)	
Hematocrit (%)	Mean ± SD	40.70 ± 4.43	40.28 ± 4.51	-0.43 (-0.45 to -0.40)	41.07 ± 4.42	42.49 ± 4.85	1.42 (1.39 to 1.44)
	Median (IQR)	40.80 (37.75, 43.70)	40.40 (37.37, 43.35)		41.13 (38.10, 44.10)	42.65 (39.30, 45.80)	
**Males, n (%)**							
Hemoglobin (g/dL)	Mean ± SD	13.92 ± 1.57	13.70 ± 1.60	-0.22 (-0.23 to -0.21)	14.00 ± 1.51	14.36 ± 1.65	0.36 (0.35 to 0.37)
	Median (IQR)	14.10 (12.95, 15.00)	13.90 (12.75, 14.80)		14.10 (13.08, 15.03)	14.50 (13.40, 15.50)	
Hematocrit (%)	Mean ± SD	42.34 ± 4.39	41.85 ± 4.52	-0.49 (-0.52 to -0.45)	42.73 ± 4.23	44.22 ± 4.70	1.49 (1.45 to 1.53)
	Median (IQR)	42.70 (39.70, 45.30)	42.28 (39.20, 44.90)		43.00 (40.13, 45.55)	44.60 (41.45, 47.45)	
**Females, n (%)**							
Hemoglobin (g/dL)	Mean ± SD	12.58 ± 1.32	12.43 ± 1.34	-0.15 (-0.16 to -0.14)	12.58 ± 1.32	12.92 ± 1.44	0.33 (0.32 to 0.35)
	Median (IQR)	12.67 (11.73, 13.50)	12.50 (11.60, 13.35)		12.65 (11.70, 13.50)	13.00 (12.00, 13.90)	
Hematocrit (%)	Mean ± SD	38.91 ± 3.72	38.55 ± 3.81	-0.36 (-0.39 to -0.33)	39.02 ± 3.74	40.35 ± 4.14	1.33 (1.29 to 1.36)
	Median (IQR)	39.05 (36.55, 41.40)	38.75 (36.20, 41.10)		39.13 (36.62, 41.50)	40.57 (37.70, 43.15)	

DPP-4is – dipeptidyl peptidase-4 inhibitors; SGLT2is – sodium–glucose cotransporter-2 inhibitors.

© 2025 Lewis M et al. *JAMA Network Open*.

eTable 8. Hemoglobin (g/dL) and Hematocrit (%) Levels Among Patients With Type 2 Diabetes, Before and After Initiating SGLT2is vs GLP-1RAs, After Propensity Score Matching

		GLP-1RAs			SGLT2is		
**Group**	**Characteristic**	**Before** N = 44,153	**After** N = 44,153	**Difference (95% CI)**	**Before** N = 42,664	**After** N = 42,664	**Difference (95% CI)**
**All patients, n (%)**							
Hemoglobin (g/dL)	Mean ± SD	13.38 ± 1.56	13.37 ± 1.55	-0.01 (-0.02 to 0.00)	13.36 ± 1.58	13.72 ± 1.70	0.37 (0.36 to 0.38)
	Median (IQR)	13.40 (12.33, 14.45)	13.40 (12.33, 14.40)		13.40 (12.30, 14.45)	13.80 (12.60, 14.90)	
Hematocrit (%)	Mean ± SD	41.12 ± 4.31	41.04 ± 4.34	-0.08 (-0.10 to -0.06)	41.02 ± 4.37	42.52 ± 4.81	1.50 (1.48 to 1.53)
	Median (IQR)	41.15 (38.20, 44.05)	41.10 (38.20, 44.00)		41.05 (38.10, 44.00)	42.65 (39.40, 45.80)	
**Males, n (%)**							
Hemoglobin (g/dL)	Mean ± SD	14.13 ± 1.49	14.08 ± 1.51	-0.05 (-0.06 to -0.04)	14.06 ± 1.48	14.45 ± 1.62	0.39 (0.38 to 0.40)
	Median (IQR)	14.30 (13.23, 15.15)	14.20 (13.20, 15.10)		14.20 (13.17, 15.10)	14.60 (13.50, 15.55)	
Hematocrit (%)	Mean ± SD	43.07 ± 4.17	42.93 ± 4.26	-0.14 (-0.17 to -0.11)	42.86 ± 4.16	44.46 ± 4.62	1.6 (1.57 to 1.64)
	Median (IQR)	43.38 (40.55, 45.87)	43.20 (40.40, 45.75)		43.10 (40.33, 45.65)	44.80 (41.80, 47.60)	
**Females, n (%)**							
Hemoglobin (g/dL)	Mean ± SD	12.70 ± 1.29	12.73 ± 1.29	0.03 (0.02 to 0.04)	12.60 ± 1.30	12.94 ± 1.42	0.35 (0.33 to 0.36)
	Median (IQR)	12.78 (11.90, 13.60)	12.80 (11.90, 13.60)		12.68 (11.75, 13.50)	13.03 (12.06, 13.90)	
Hematocrit (%)	Mean ± SD	39.40 ± 3.65	39.38 ± 3.69	-0.02 (-0.05 to 0.00)	39.05 ± 3.68	40.44 ± 4.10	1.39 (1.36 to 1.43)
	Median (IQR)	39.50 (37.06, 41.85)	39.50 (37.00, 41.80)		39.15 (36.70, 41.50)	40.67 (37.87, 43.20)	

GLP-1RAs – glucagon-like peptide-1 receptor agonists; SGLT2is – sodium–glucose cotransporter-2 inhibitors.

© 2025 Lewis M et al. *JAMA Network Open*.

eTable 9. Hemoglobin (g/dL) and Hematocrit (%) Levels Among Patients With Type 2 Diabetes Before and After Initiating SGLT2is (Unmatched Cohort), Stratified by SGLT2i Type

		Dapagliflozin			Empagliflozin		
**Group**	**Characteristic**	**Before** N = 18,995	**After** N = 18,995	**Difference (95% CI)**	**Before** N = 40,855	**After** N = 40,855	**Difference (95% CI)**
Hemoglobin (g/dL)	Mean ± SD	13.31 ± 1.62	13.63 ± 1.74	0.33 (0.31 to 0.34)	13.46 ± 1.60	13.81 ± 1.73	0.35 (0.34 to 0.36)
	Median (IQR)	13.30 (12.20, 14.45)	13.70 (12.48, 14.85)		13.50 (12.40, 14.60)	13.90 (12.70, 15.00)	
Hematocrit (%)	Mean ± SD	40.88 ± 4.53	42.16 ± 4.95	1.3 (1.2 to 1.3)	41.34 ± 4.43	42.78 ± 4.87	1.4 (1.4 to 1.5)
	Median (IQR)	40.90 (37.80, 43.93)	42.30 (38.90, 45.55)		41.40 (38.37, 44.40)	42.95 (39.60, 46.15)	

SGLT2is – sodium–glucose cotransporter-2 inhibitors.

© 2025 Lewis M et al. *JAMA Network Open*.

eTable 10. Cox Proportional Hazards Regression Model With Time-Varying Exposure for Myocardial Infarction, Venous Thromboembolism, and Stroke Comparing Patients With Type 2 Diabetes After Initiating SGLT2is and Developing New-Onset Erythrocytosis (Cohort 3)

	**Hazard Ratio (95% Confidence Interval)**			
**Outcome**	**Any Thrombotic Event**	**Myocardial Infarction**	**Venous Thromboembolism**	**Stroke**
Erythrocytosis (Strict criteria, model 1)	1.12 (0.84 to 1.49)	0.92 (0.58 to 1.44)	1.56 (0.68 to 3.59)	1.26 (0.84 to 1.89)
Erythrocytosis (Lenient criteria, model 2)	0.72 (0.57 to 0.92)	0.73 (0.52 to 1.02)	0.90 (0.39 to 2.10)	0.68 (0.47 to 0.99)

SGLT2i – sodium–glucose cotransporter-2 inhibitor.

The models were also adjusted for sex, age, smoking status, comorbidities, SGLT2i type, and glomerular filtration rate.

Lenient criteria: hemoglobin>16.5 g/dL or hematocrit >49% in males, and hemoglobin>16.0 g/dL or hematocrit>48% in females. Based on the 2016 WHO classification.

Strict criteria: hemoglobin >18.5 g/dL or hematocrit ≥52% in males, and hemoglobin>16.5 g/dL or hematocrit≥48% in females. Based on the 2005 British Society of Hematology criteria.

© 2025 Lewis M et al. *JAMA Network Open*.

eTable 11. Coding and Definitions

**Diagnoses**	**Codes**
Hypertension	ICD-9: 401-405
Myocardial infarction	ICD-9: 410.xx<br>ICD-10: I21.xx
Stroke	ICD-9: 430.xx, 431.xx, 433.x1 (*excluding 433.x0), 434.x1 (excluding 434.x0), 436.xx<br>*Carotid artery stenosis/carotid plaque were excluded
Ischemic heart disease	ICD-10: I25 (excluding I25.3)
Heart failure	ICD-9: 428.X
Peripheral vascular disease	ICD-9: 443 (excluding 443.2)<br>ICD-10: I73
Venous thromboembolism	ICD-9: 415.1, 415.11, 415.19,453.2, 453.3, 453.8, 453.9, 453.4, 453.41, 453.42, 453.40, 453.41, 453.82, 453.83, 453.84, 453.85, 453.89
Atrial fibrillation	ICD-9: 427.3x
Chronic kidney disease	ICD-9: 585.X
Obstructive lung diseases	ICD-10: J44, J45
Obstructive sleep apnea	ICD-9: 327.23
Liver diseases	ICD-9: 571.x
Malignancy	140.x-172.x, 174.x-195.x, 200.x-202.x, 203.0, 238.6
**Exclusion criteria**	
Familial polycythemia (ever before)	ICD-9 code 289.6
Secondary erythrocytosis (in the previous year)	ICD-9 code 289.0
Polycythemia Vera (ever before)	ICD-9 code 238.4
Essential thrombocytosis (ever before)	ICD-9 code 238.71
Myelofibrosis (ever before)	ICD-9 code 238.76
**Medications**	
Metformin	ATC: A10BA02
Sodium–glucose cotransporter-2 inhibitor	ATC: A10BK
Dipeptidyl peptidase-4 inhibitor	ATC: A10BH

© 2025 Lewis M et al. *JAMA Network Open*.
