# Supplementary material for: Sodium-Glucose Cotransporter 2 Inhibitors, Erythrocytosis, and Thrombosis in Adults With Type 2 Diabetes
Source: JAMA Netw Open. 2025 Jun 23;8(6):e2517086. doi: 10.1001/jamanetworkopen.2025.17086 (PMC12186121; doi:10.1001/jamanetworkopen.2025.17086)
Supplement: Supplement 2. — Data Sharing Statement [file jamanetwopen-e2517086-s002.pdf]

Data Sharing Statement

Lewis. Sodium-Glucose Cotransporter 2 Inhibitors, Erythrocytosis, and Thrombosis in Adults With Type 2 Diabetes. *JAMA Netw Open*. Published June 23, 2025. doi:10.1001/jamanetworkopen.2025.17086

Data
Data available: No

Additional Information
Explanation for why data not available: The data that support the findings of this study originate from a large Israeli HMO. Restrictions apply to the availability of these data and they are therefore not publicly available.
